# Production of transgenic pig as an Alzheimer’s disease model using a multi-cistronic vector system

**DOI:** 10.1371/journal.pone.0177933

**Published:** 2017-06-06

**Authors:** Seung-Eun Lee, Hyuk Hyun, Mi-Ryung Park, Youngsok Choi, Yeo-Jin Son, Yun-Gwi Park, Sang-Gi Jeong, Min-Young Shin, Hee-Jin Ha, Hyun-Sok Hong, Min-Keyung Choi, Gi-Sun Im, Eung-Woo Park, Young-Ho Kim, Chankyu Park, Eun-Young Kim, Se-Pill Park

**Affiliations:** 1 Faculty of Biotechnology, College of Applied Life Sciences, Jeju National University, Jeju-si, Jeju Special Self-Governing Province, Korea; 2 Stem Cell Research Center, Jeju National University, Jeju-si, Jeju Special Self-Governing Province, Korea; 3 Animal Biotechnology Division, National Institute of Animal Science, Rural Development Administration, Wanju-gun, Jeollabuk-do, Korea; 4 Department of Biomedical Science, CHA University, Pocheon-si, Gyeonggi-do, Korea; 5 Medifron DBT, Ansan-si, Gyeonggi-do, Korea; 6 Department of Animal Biotechnology, Konkuk University, Seoul, Korea; 7 Mirae Cell Bio, Seoul, Korea; Torrey Pines Institute for Molecular Studies, UNITED STATES

## Abstract

Alzheimer’s disease (AD) is a progressive neurodegenerative disease associated with memory loss and cognitive impairments. An AD transgenic (Tg) pig model would be useful for preclinical testing of therapeutic agents. We generated an AD Tg pig by somatic cell nuclear transfer (SCNT) using a multi-cistronic vector that harbored three AD-related genes with a total of six well-characterized mutations: hAPP (K670N/M671L, I716V, and V717I), hTau (P301L), and hPS1 (M146V and L286P). Four AD Tg cell lines were established from Jeju black pig ear fibroblasts (JB-PEFs); the resultant JB-PEF^AD^ cells harbored transgene integration, expressed transgene mRNAs, and had normal karyotypes. Tg line #2–1, which expressed high levels of the transgenes, was used for SCNT; cleavage and blastocyst rates of embryos derived from this line were lower than those of Non-Tg. These embryos yielded three piglets (Jeju National University AD-Tg pigs, JNUPIGs) revealed by microsatellite testing to be genetically identical to JB-PEF^AD^. Transgenes were expressed in multiple tissues, and at especially high levels in brain, and Aβ-40/42, total Tau, and GFAP levels were high in brains of the Tg animals. Five or more copies of transgenes were inserted into chromosome X. This is the first report of an AD Tg pig derived from a multi-cistronic vector.

## Introduction

Alzheimer’s disease (AD), the most common cause of dementia, accounts for approximately 60–70% of dementia cases and afflicts more than 35.6 million individuals worldwide; this number is predicted to increase to 65.7 million by 2030 and 115.4 million by 2050 [1]. In addition to being a serious public health problem, AD also increases the cost of medical care around the world.

AD is a relentlessly progressive disorder that typically initially manifests as severe loss of memory, particularly episodic memory. At present, the disorder is not curable, increasing the urgency of developing and characterizing relevant AD transgenic (Tg) animal models to facilitate translational research and preclinical testing of therapeutic agents [2]. Animal models are critical tools for drug development and experimental medical science because they contribute to improved understanding of the pathogenesis of human diseases. An enormous amount of preclinical evidence in animal models has been required for further clinical development of pharmacological drugs that can interfere with most of the damaged neuronal pathways in AD patients [3]. Once developed, such models can be exploited to test therapeutic strategies for treating the functional disturbances associated with the disease of interest [4–6].

Several species, in particular mice, have been used to create genetically altered phenocopies of human AD. To date, however, no study has reported a pig model that can fully reproduce the features of disease progression in sporadic/late-onset AD, which represents the vast majority of AD cases [7]. Mice have been extensively used as AD Tg models, and the resultant work has expanded our understanding of the molecular mechanisms associated with amyloid beta (Aβ) production [8]. For example, the amyloid precursor protein (APP) Tg mouse model develops extensive parenchymal and vascular amyloid deposits similar to those of human AD [9]. Although such AD Tg mice provide value`ble information regarding the role of inflammation, the progressive neuronal loss in the hippocampus and specific neocortical regions of the human AD brain is not evident in most of these models [10], underscoring the limited utility of rodent systems for mimicking human disease. Compared with mice, pigs are more similar to humans regarding anatomy, neurobiology, longevity, and genetics, and accordingly porcine models have been used successfully to model human diseases. Pigs have long life spans, are easily bred, and reach puberty within 5–6 months; moreover, for ethical and economic reasons they are preferable to other large animals, such as primates, as biomedical research subjects [11].

AD is defined clinically by a gradual decline in memory and other cognitive functions, and neuropathologically by gross atrophy of the brain and accumulation of extracellular amyloid plaques and intracellular neurofibrillary tangles [12]. APP, tau protein, and Presenilin 1 (PS1) are hallmarks of damaged neurons in AD patients. Aβ is a proteolytic fragment of APP [13] generated by sequential cleavage of precursor by β- and γ-secretases [14]. PS1 is the sub-component of γ-secretases responsible for the cutting of APP [15]. Deposition of extracellular amyloid plaques is followed by accumulation of neurofibrillary tangles, consisting of hyper-phosphorylated tau aggregates, in neuronal cell bodies and associated processes [16]. Dominant mutations in APP, tau protein, and PS1 cause inherited (familial) early-onset AD. Therefore, accumulation of Aβ and formation of neurofibrillary tangles are essential features of an effective AD animal model. However, no previous study has reported an AD Tg pig model that simultaneously expresses the major AD mutant genes.

In this study, we sought to generate an AD pig model using a multi-cistronic vector harboring mutated versions of major AD-related genes. We designed such a vector encoding three AD-related genes with a total of six well-characterized mutations: hAPP (K670N/M671L, I716V, and V717I), hTau (P301L), and hPS1 (M146V and L286P). Expression of these mutant genes was predicted to cause AD in Tg pigs by promoting aggregation of Aβ and hyper-phosphorylated tau protein in the brain. In four stable AD Tg cell lines derived by introduction of the multi-cistronic vector into Jeju black pig ear fibroblasts (JB-PEF^AD^), we examined transgene integration, transgene mRNA expression, and karyotype. In addition, we monitored SCNT^AD^ embryo development and transgene mRNA expression in individual blastocysts. Three piglets (Jeju National University AD-Tg pigs, JNUPIGs) were evaluated for expression of transgene and by microsatellite test; two of these JNUPIGs were born live, whereas the third was sacrificed and used to assess transgene expression in multiple tissues. We confirmed that transgenes were expressed at higher levels in brain than in other tissues, and also characterized Aβ-40/42 levels in brain, identified the insertion sites, and determined transgene copy numbers. The resultant Tg pigs could be used as a large-animal model for testing of experimental therapeutics against AD.

## Results

### Construction of the AD multi-cistronic vector

**Fig 1:**
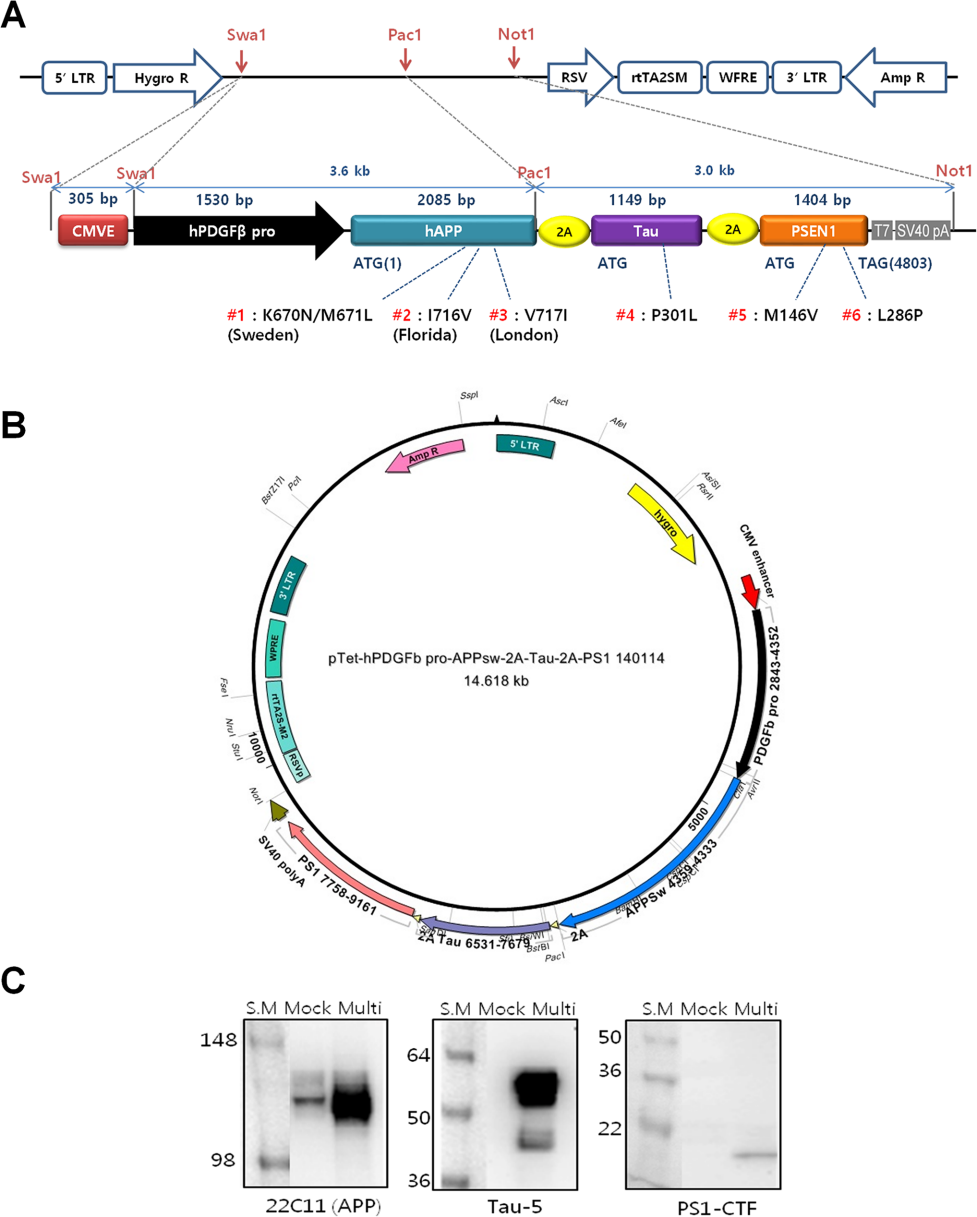
Structure of pTet retroviral multi-cistronic vector containing hAPP, hTau, and hPS1 under the control of the CMVE and hPDGFβ promoter. (A) Linear map. (B) Circular map. (C) Protein expression of AD transgenes in HEK cells. CMVE, human CMV enhancer; hPDGFβ, human platelet–derived growth factor β; hAPP, human amyloid precursor protein; hTau, human tau protein; hPS1, human presenilin 1. Three different gene product were detected by Western blot analysis with antibody against hAPP (22C11), Tau (Tau-5), PS1 (PS1-CTF). S.M: protein size marker, Mock and Multi; control cell and multi-cistronic vector transfected cell, repectively.

We generated the multi-cistronic vector by inserting hAPP, hTau, and PS1 genes under control of the hPDGFβ promoter into a modified pTet-CKOS retroviral vector using the primers (Fig 1A and Table 1). A fragment of the CMV enhancer (CMVE) was inserted upstream of the hPDGFβ promoter to increase transgene expression in neurons [8]. A circular map of the final construct is shown in Fig 1B, and information about its full sequence is provided in S1 Fig. Transgene expression was first verified by transient transfection into HEK cell lines. Western blot analysis was confirmed using specific antibodies against hAPP, Full length Tau and activated PS1 (Fig 1C). For detection of human APP, we used antibodies 22C11 (vs. hAPP N-terminus). These experiments revealed that full-length APP was successfully expressed in HEK293 cells. Human Tau and PS1 were detected using antibodies against hTau (Tau5 antibody) and PS1-CTF (anti-PS1 antibody), respectively.

**Table 1:** Oligonucleotides for cloning and mutagenesis.

Target	Primer name	Sequence (5'->3')	Size (bp)
pTet-enz.	pTet-Enz-insert	F:AGATCTATTTAAATACCGGT	67
insert	pTet-Enz-insert	R:CTCGAGGCGGCCGCCCTGCA	
hPDGFb	BamH1-Swa1-PDGFb	F:GGATCCATTTAAATGCTGGGACTACAGGAGCTTG	1,530
promoter	PDGFb-Cla1	R:ATCGATGTGCGCGCAAAGTATCTCTA	
hAPP cDNA	Cla1-hAPPsw	F:ATCGATATGCTGCCCGGTTTGGCACT	2,106
	hAPPsw-Pac1-Sph1	R:GGCATGCTTAATTAAGTTCTGCATCTGCTCAAAGA	
hAPP	hAPPsw-M1(KM/NL)	F:GAGATCTCTGAAGTG AATCTGGATGCAGAATTCCGA	-
Mutagenesis-1	hAPPsw-M1(KM/NL)	R:TCGGAATTCTGCATCCAGATTCACTTCAGAGATCTC	-
hAPP	hAPPsw-M2(IV/VI)	F:GTCATAGCGACAGTGGTCATCATCACCTTGGTGATG	-
Mutagenesis-2	hAPPsw-M2(IV/VI)	R:CATCACCAAGGTGATGATGACCACTGTCGCTATGAC	-
hTau cDNA	Bgl2-hTau	F:AGATCTATGGCTGAGCCCCGCCAGGA	1,161
	hTau-EcoR1	R:GAATTCCAAACCCTGCTTGGCCAGGG	
hTau	hTau-M(P/L)	F:AATATCAAACACGTCCTGGGAGGCGGCAGTGTGC	-
Mutagenesis	hTau-M(P/L)	R:CACACTGCCGCCTCCCAGGACGTGTTTGATATT	-
2A peptide	EcoR1-2A	F:GAATTCGGAAGCGGAGCTACTAACTT	78
	2A-Xho1	R:CTCGAGAGGTCCAGGGTTCTCCTCCA	
hPS1 cDNA	Xho1-hPS1	F:CTCGAGATGACAGAGTTACCTGCACC	1,424
	hPS1-Xba1	R:TCTAGACCTGCAGGCTAGATATAAAATTGATGGA	
hPS1	hPS1-M1(M/V)	F:AGTGTCATTGTTGTCCTGACTATCCTCCTGGTG	-
Mutagenesis-1	hPS1-M1(M/V)	R:CACCAGGAGGATAGTCAGGACAACAATGACACT	-
hPS1	hPS1-M2(L/V)	F:TGAAACGCTTTTTCCAGCTGTCATTTACTCCTCAACA	-
Mutagenesis-2	hPS1-M2(L/V)	R:TGTTGAGGAGTAAATGACAGCTGGAAAAAGCGTTTCA	-
CMV enhancer	CMVE	F:ATTTAAATGCGTTACATAACTTACGG	-
	CMVE	R:ATTTAAATCATGGTAATAGCGATGAC	-

* F, forward; R, reverse.

### Generation of AD Tg cell lines

**Fig 2:**
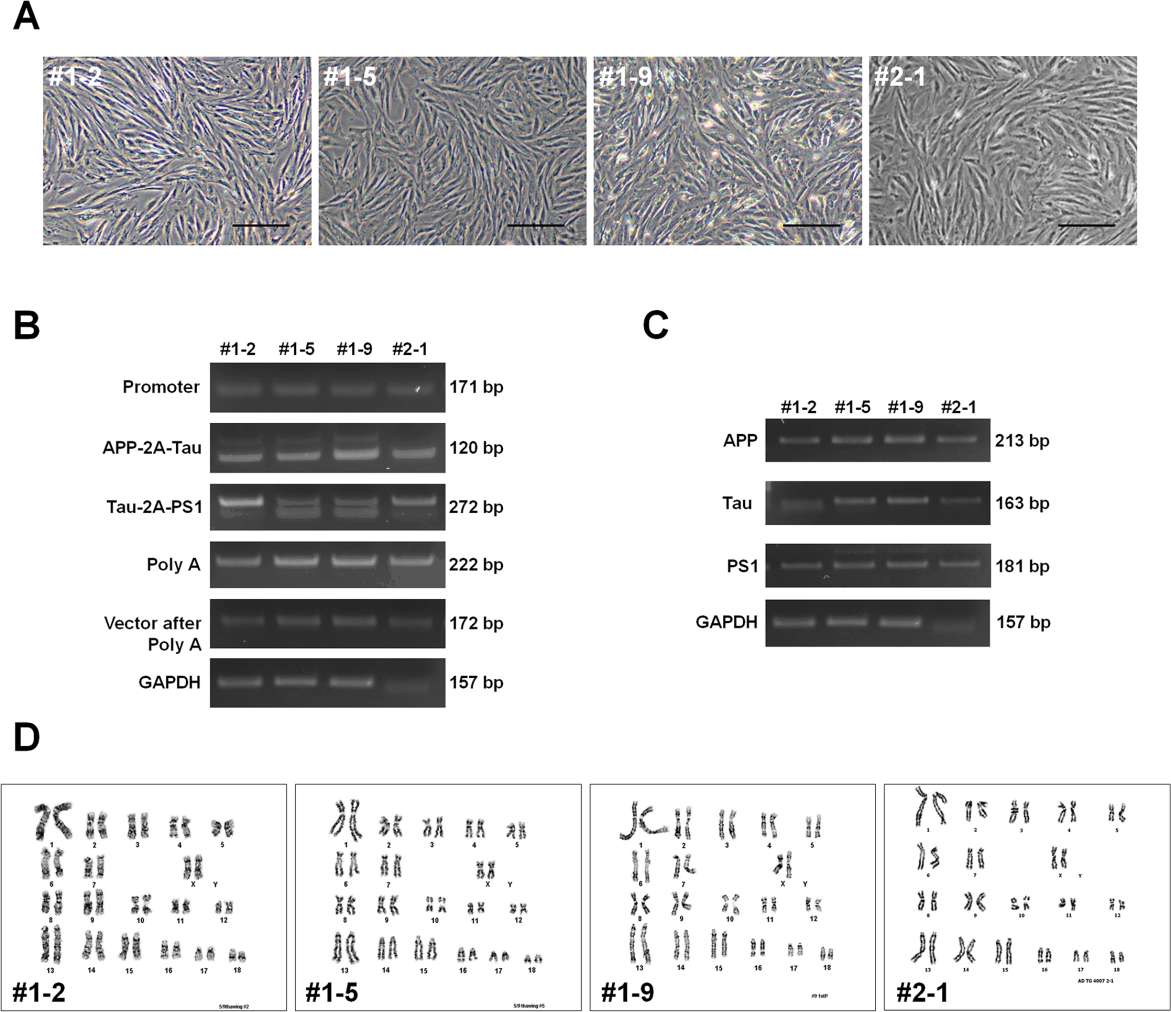
Preparation of AD transgenic Jeju black pig ear fibroblasts (JB-PEF^AD^). Morphology (A), integration (B), and mRNA expression (C) of AD multi-cistronic vector in JB-PEF^AD^ cells. Karyotype (D) of JB-PEF^AD^ cell lines, revealing normal chromosome number (2n = 38). AD multi-cistronic vector: hAPP, human amyloid precursor protein; hTau, human tau protein; PS1, presenilin 1. Scale bars = 20 μm.

AD Tg Jeju black pig ear fibroblast (JB-PEF^AD^) lines were established by transfection of linearized retroviral multi-cistronic vector containing hAPP, hTau, and hPS1 under the control of a fusion promoter (CMVE+ hPDGFβ promoter region), the SV40 poly A site, and a hygromycin resistance gene. Transfections were conducted either using a lipofection reagent or by electroporation. Stable transfected cells were selected by culture in hygromycin B (40 ng/mL) for 12 days, followed by colony selection and picking. We established four Tg cell lines (Fig 2A; #1–2, #1–5, #1–9, and #2–1) and used polymerase chain reaction (PCR) to confirm chromosomal integration of the modified retroviral multi-cistronic vector including the fusion promoter, three mutated genes, and SV40 poly A tail (Fig 2B). All four JB-PEF^AD^ cell lines expressed hAPP, hTau, and hPS1 transcripts (Fig 2C) and had normal karyotypes compared to that of normal cells (Fig 2D).

### Development and characterization of SCNT^AD^ embryos

**Fig 3:**
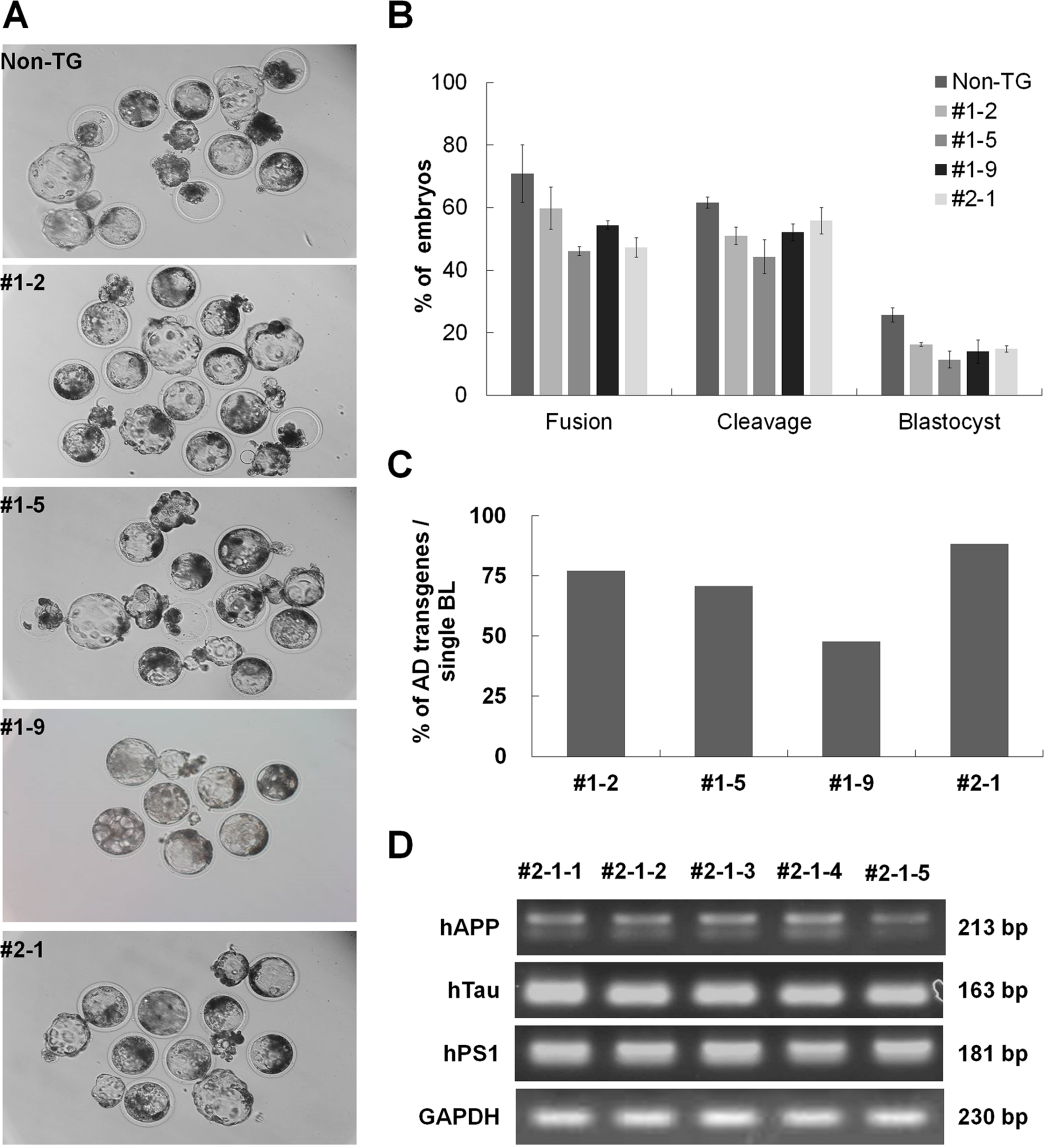
**Morphology (A) of blastocysts derived from SCNT**^**AD**^ **embryos on day 7, and developmental rate (B) of SCNT**^**AD**^ **embryos.** The gDNAintegrationrates (C) of AD transgenes in individual blastocysts, determined by PCR. Expression levels (D) in five blastocysts derived from SCNT embryos using transgenic cell line #2-1.hAPP, human amyloid precursor protein; hTau, human tau protein; PS1, presenilin 1. Scale bars = 120 μm.

We then performed SCNT with JB-PEF^AD^ cells to generate SCNT^AD^ embryos. The morphology of blastocysts derived from non-TG, #1–2, #1–5, #1–9, and #2–1 JB-PEF^AD^ cell lines is shown in Fig 3A. The fusion rates, cleavage rates, and blastocyst formation rates of SCNT^AD^ embryos derived from the four Tg and one non-Tg cell lines were 59.8±6.7%, 46.2±1.4%, 54.4±1.4%, 47.3±3.1%, and 70.9±9.2 (Fig 3B); 51.0±2.8%, 44.3±5.4%, 52.1±2.7%, 55.8±4.3%, and 61.6±1.8%; and 16.3±0.6%, 11.4±2.7%, 14.0±3.7%, 14.8±1.1%, and 25.7±2.2%, respectively (Fig 3B). *In vitro* development ability was lower in all four Tg cell lines than in the non-Tg line, but did not differ significantly among the Tg lines. The genomicDNA (gDNA) integration rates of AD transgenes on #1–2, #1–5, #1–9, and #2–1 were 77.1%, 70.7%, 47.8%, and 88.4%, respectively (Fig 3C); thus transgene expression was highest for line #2–1. Furthermore, all blastocysts of Tg cell line#2–1 contained DNA encoding hAPP, hTau, and hPS1, as determined by PCR (Fig 3D).

### Production and analysis of AD transgene—expressing cloned fetuses

**Fig 4:**
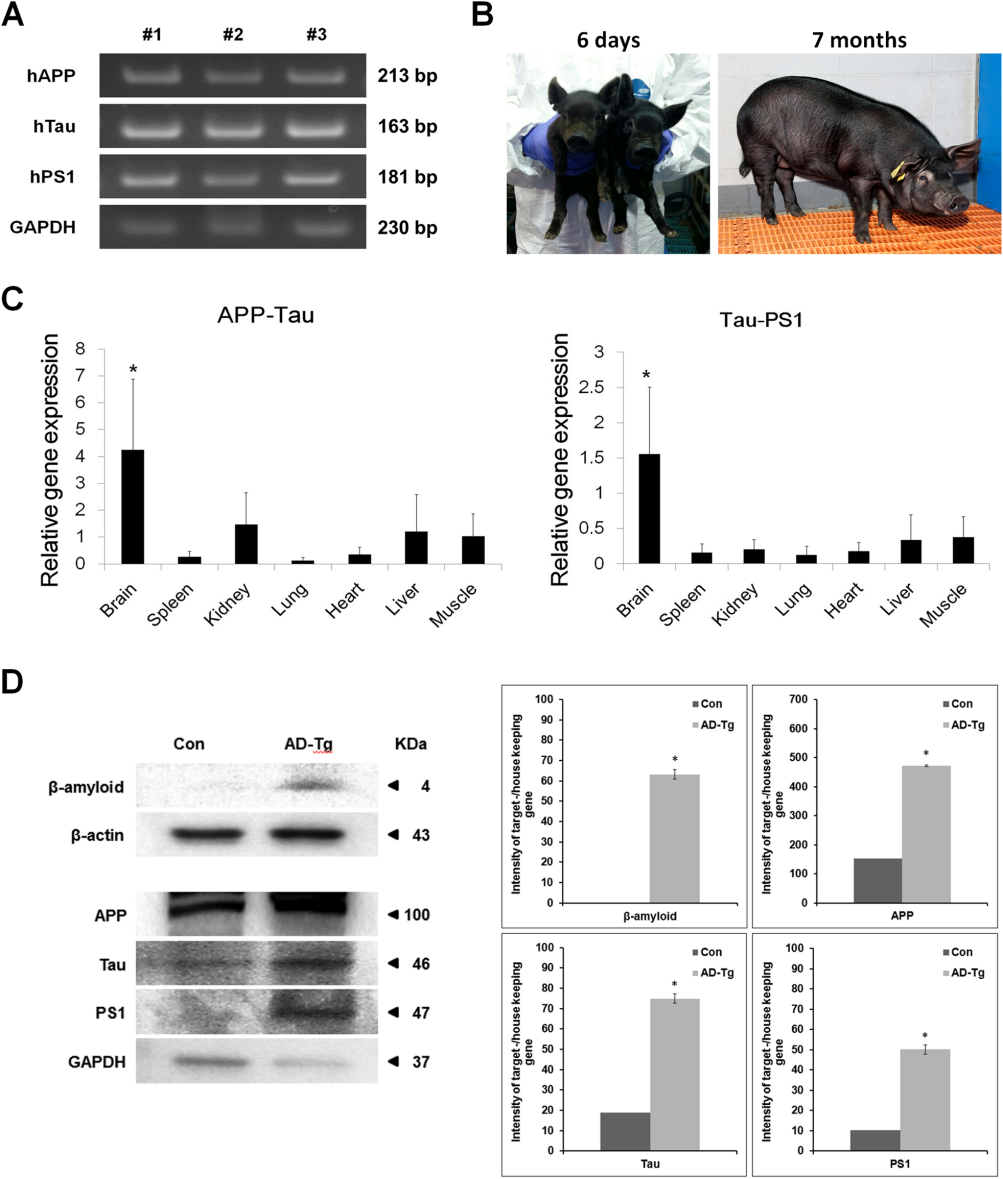
**Production of AD transgenic cloned pigs and identification of AD transgenes (A), hAPP, hTau, and hPS1 in cloned pigs: #1, 2 (alive, B), and 3 (stillborn).** Expression of APP-Tau and Tau-PS1 in the tissues of AD transgenic pig, determined by real-time RT-PCR (C) and expression of AD-related genes in brain of TG pigs (D). hAPP, human amyloid precursor protein; hTau, human tau protein; hPS1: human presenilin 1. The experiment was repeated three or four independent times.

SCNT was performed using JB-PEF^AD^#2–1, and 1,834 embryos were transferred into the oviducts of 11 recipient gilts (Table 2). Three recipients (27.3%) became pregnant, as determined by ultrasound 28 days post-transfer. Two recipients (18.2%) maintained pregnancy to term, and both delivered via natural birth 118 days after embryo transfer (ET) (Table 3). One sow delivered a single piglet without AD transgenes that died soon after being born. The other sow delivered three JNUPIGs with all three AD transgenes (hAPP, hTau, and hPS1) (Fig 4A), one of which was stillborn. The live JNUPIGs weighed 1,130 g and 820 g at 6 days and 76 kg at 7 months after birth, exhibited normal morphology (Fig 4B), and had all three transgenes as determined assessed by PCR (Fig 4A). Genetic analysis of 14 polymorphic microsatellite loci confirmed that the three JNUPIGs were identical to the JB-PEF^AD^ cell used for nuclear transfer (Table 4). One of the two live-born JNUPIGs was sacrificed for assays at 70 days after birth. We analyzed hAPP-hTau and hTau-hPS1 by real-time RT-PCR in multiple tissues of this JNUPIG (Fig 4C). The results revealed that the transgenes were expressed at significantly higher levels in brain than in other tissues (p<0.05). We checked β-amyloid, APP, Tau, PS1 from brain tissue of JNUPIG and control pig by western blotting (Fig 4D). The results indicated that the transgenes were expressed at significantly higher levels in JNUPIG than in control pig (p<0.05).

**Table 2:** Production of AD transgenic piglets.

No. of embryos transferred	No. (%) of recipient			No. of cloned piglets	Stillbirt	Live birth	No. (%) of transgenic piglets	Weight* (kg)
	Day 30 pregnancy	Day 90 pregnancy	delivered					
1,834	3/11 (27.3)	2/11 (18.2)	2/11 (18.2)	3	1	2	2/3 (66.7)	1.01±0.16

* Weights of piglets are expressed as means **±** SEMs

**Table 3:** Full-term development of AD transgenic piglets.

ET no.	Recipients	No. of transferred embryos (NT+PA)	Day 30 pregnancy status*	Day 90 pregnancy status	No. of piglets at birth (transgenic)	Specificity
**1**	**30–03**	**225 (110+115)**	**-**	**-**		
**2**	**30–04**	**238 (120+118)**	**+**	**+**	**1 (0)**	**Died after birth**
**3**	**30–32**	**202 (152+50)**	**+**	**+**	**3 (3)**	**1 stillbirth**
**4**	**30–33**	**156 (106+50)**	**-**	**-**		**2 live births**
**5**	**30–63**	**146 (95+61)**	**+**	**-**		
**6**	**30–66**	**152 (152+0)**	**-**	**-**		
**7**	**30–71**	**86 (86+0)**	**-**	**-**		
**8**	**30–72**	**102 (102+0)**	**-**	**-**		
**9**	**30–78**	**215 (215+0)**	**-**	**-**		
**10**	**30–08**	**157 (107+50)**	**-**	**-**		
**11**	**30–10**	**155 (106+49)**	**-**	**-**		

* +, Pregnant; -, not pregnant.

**Table 4:** Microsatellite (MS) analysis of donor surrogate, donor cell, and offspring.

No. of MS markers*	Surrogate mother		Donor cell		TG #1		TG #2		TG #3	
	Peak 1	Peak 2	Peak 1	Peak 2	Peak 1	Peak 2	Peak 1	Peak 2	Peak 1	Peak 2
S02276	190	198	190	190	190	190	190	190	190	190
SW210	235	237	237	237	237	237	237	237	237	237
SW2410	106	106	106	118	106	118	106	118	106	118
S0107	188	196	196	196	196	196	196	196	196	196
S0277	227	239	239	239	239	239	239	239	239	239
S0068	237	245	211	247	211	247	211	247	211	247
SW2515	93	99	93	93	93	93	93	93	93	93
SW2401	157	159	163	163	163	163	163	163	163	163
SW1989	230	246	240	244	240	244	240	244	240	244
SWR1941	214	216	218	222	218	222	218	222	218	222
SWR1849	138	152	144	146	144	146	144	146	144	146
SW1337	208	208	206	208	206	208	206	208	206	208
S0018	250	270	248	252	248	252	248	252	248	252
SW2456	204	208	190	190	190	190	190	190	190	190

* For each microsatellite marker, genotype was determined by size (base pairs). This results provide the strongest support for the genetic identity of the donor cells and the nuclear transfer piglets.

**Fig 5:**
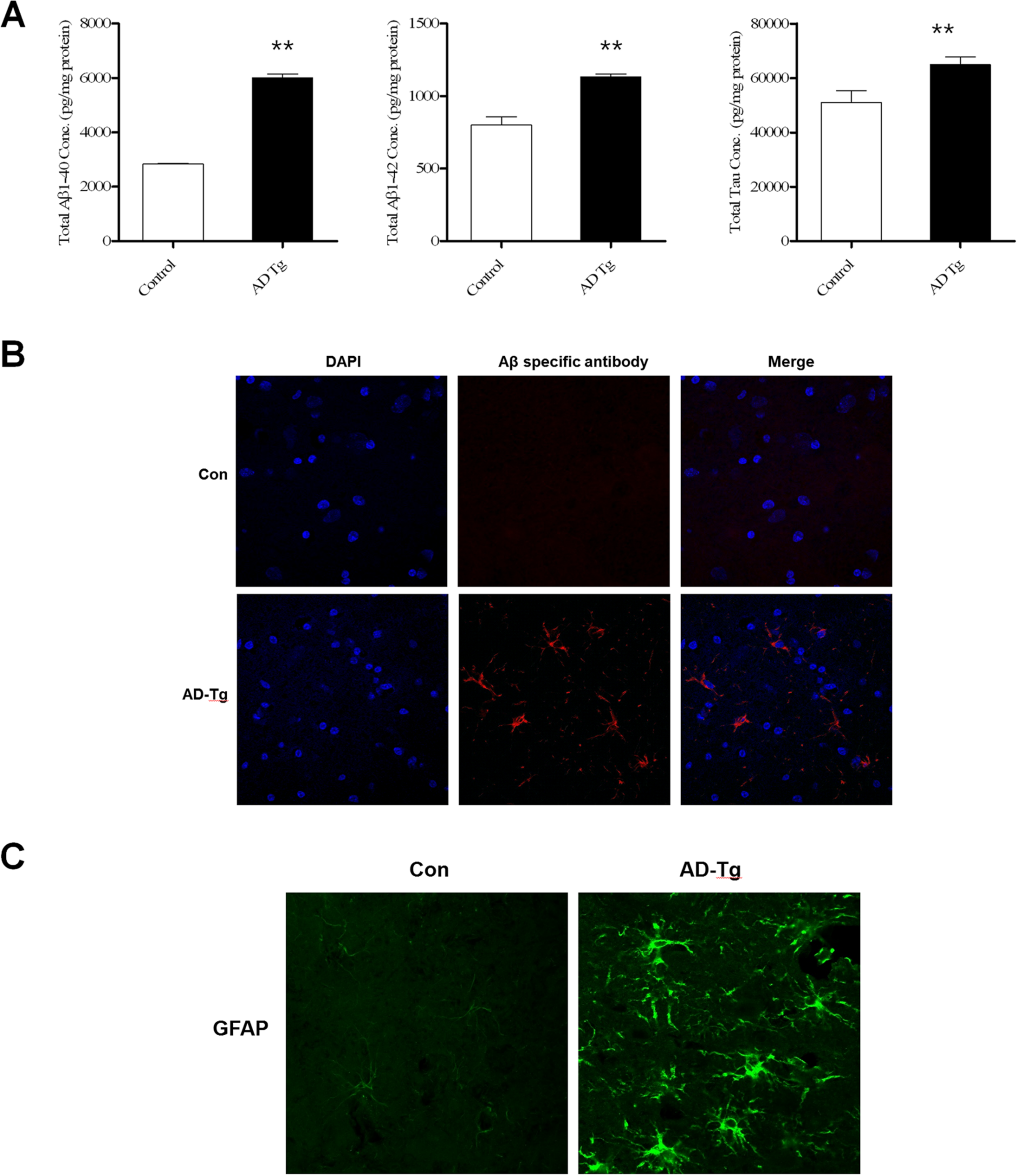
Bio-marker analysis of Tg pigs. (A) Quantification of Aβ1–40, Aβ1–42, and total Tau in the cortex of control (blank box) and Tg brains (black bar) using ELISA. All three gene expression was elevated in the TG brain. (B) Expression of APP in the cortex region of JNUPIGs confirmed by immunohistochemistry stained with anti-Aβ specific antibody. (C) Astrocyte activation, monitored by GFAP level, in control and Tg brain. GFAP level was highly elevated in TG brain. Images, taken at 400× magnification, are representative of at least four sections per animal. Data were analyzed by independent samples t-test in SPSS (*: p < 0.05, **: p < 0.01). The experiment was repeated three independent times.

We analyzed Aβ levels from brain lysates of JNUPIG and control pigs. In JNUPIG brain lysates, Aβ1–40 and Aβ1–42 levels were 6016 and 1132 pg/mg protein, respectively, whereas in control brain the levels were 2841 and 800 pg/mg protein, respectively (Fig 5A). Thus, even at very early time points, AD Tg brain contained very high levels of Aβ, roughly twice the wild-type level. Although the ELISA kit for human Aβ40/42 could cross-react with porcine Aβ40/42, it was clear that JNUPIG brain contained more Aβ than the control. The total Tau level also increased around 1.3 fold in JNUPIG brain compared to the control.

Expression of Aβ was evaluated by immunostaining with anti-Aβ antibody (Fig 5B), revealing elevated levels of Aβ in the cortex of JNUPIG. Accumulation of intracellular Aβ was only detected in JNUPIG (Fig 5B, middle lower panel), although extracellular amyloid plaques were not observed at this time point.

Astrocyte activation is a hallmark of AD because formation of Aβ plaques damages surrounding brain regions. Therefore, abnormal production of Aβ in AD Tg brain is predicted to cause astrocyte activation and inflammation in the brain. Accordingly, we checked the level of GFAP, which is secreted by activated astrocytes, using immunohistochemistry. The GFAP level was higher in JNUPIG brain than in the control (Fig 5C). Thus, it is clear that elevation of Aβ levels is closely associated with elevated secretion of GFAP.

### Determination of transgene insertion sites and copy numbers

**Fig 6:**
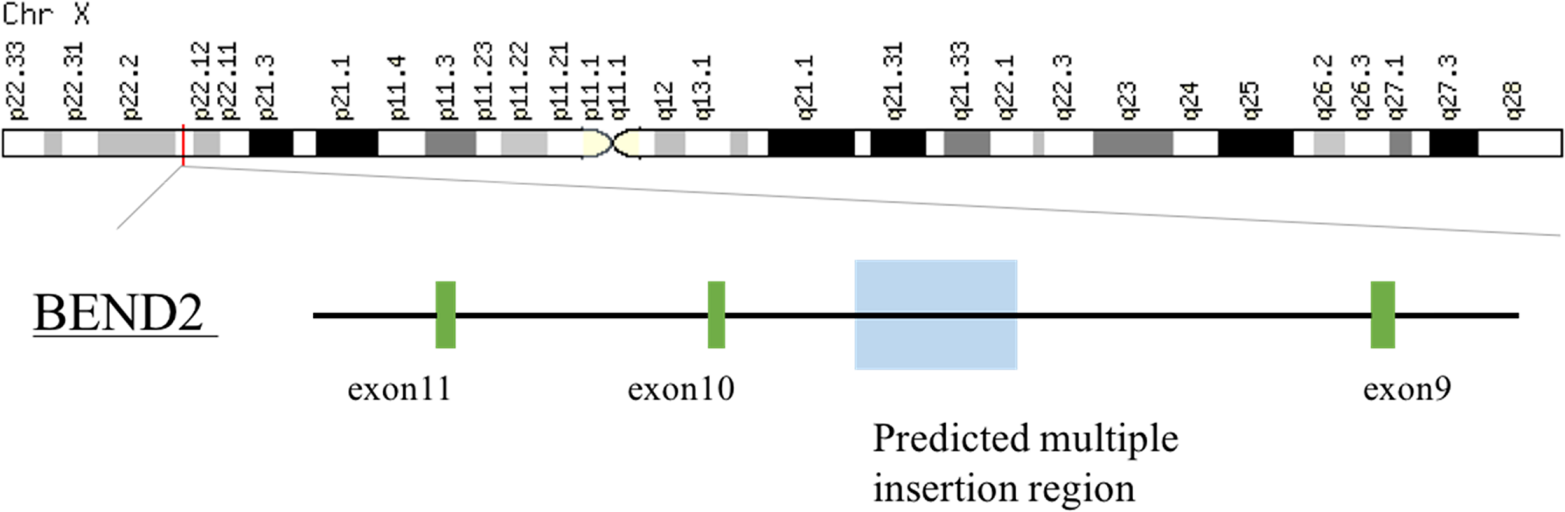
Predicted multiple insertion sites in chromosome X of transgenic pigs. The insertion site was determined by NGS analysis of gDNA of a transgenic pig. The transgene was inserted into the region between exons 9 and 10 of *BEND2*.

To characterize the positions of transgene insertions and copy numbers, we subjected the gDNA of a JNUPIG to next-generation sequencing (NGS). This analysis generated 130.2 Gbp of high-quality sequencing results (Phred quality score >30) of paired-end reads, corresponding to 46.5× genome coverage (total reads base = 139.4 giga base pairs). The reads were aligned using BLAST against both the current pig genome assembly and the 14.6 kb sequence of the transgenic vector. From the results, we identified 271 chimeric reads that matched both pig genome and Tg construct sequences. However, when we subjected the chimeric reads to stringent selection criteria including manual inspection, only the X-chromosome persisted as a valid integration site for the Tg construct (S1 Table). Because the entire circular-form construct was used for transformation without being linearized, resulting in multi-copy insertion with different configurations of the Tg construct, precise determination of the integration site was difficult. However, our analysis of chimeric reads revealed at least four distinct copies of different types of Tg inserts (starting from nucleotide positions 1697, 3709, 11314, and 11527 of the Tg vector sequence) within the *BEND2* gene between Xp22.1 and p22.2 (S1 Table and Fig 6). However, due to the complex integration structures, we were unable to determine the integrity and predict the functional activity of each insertion.

**Fig 7:**
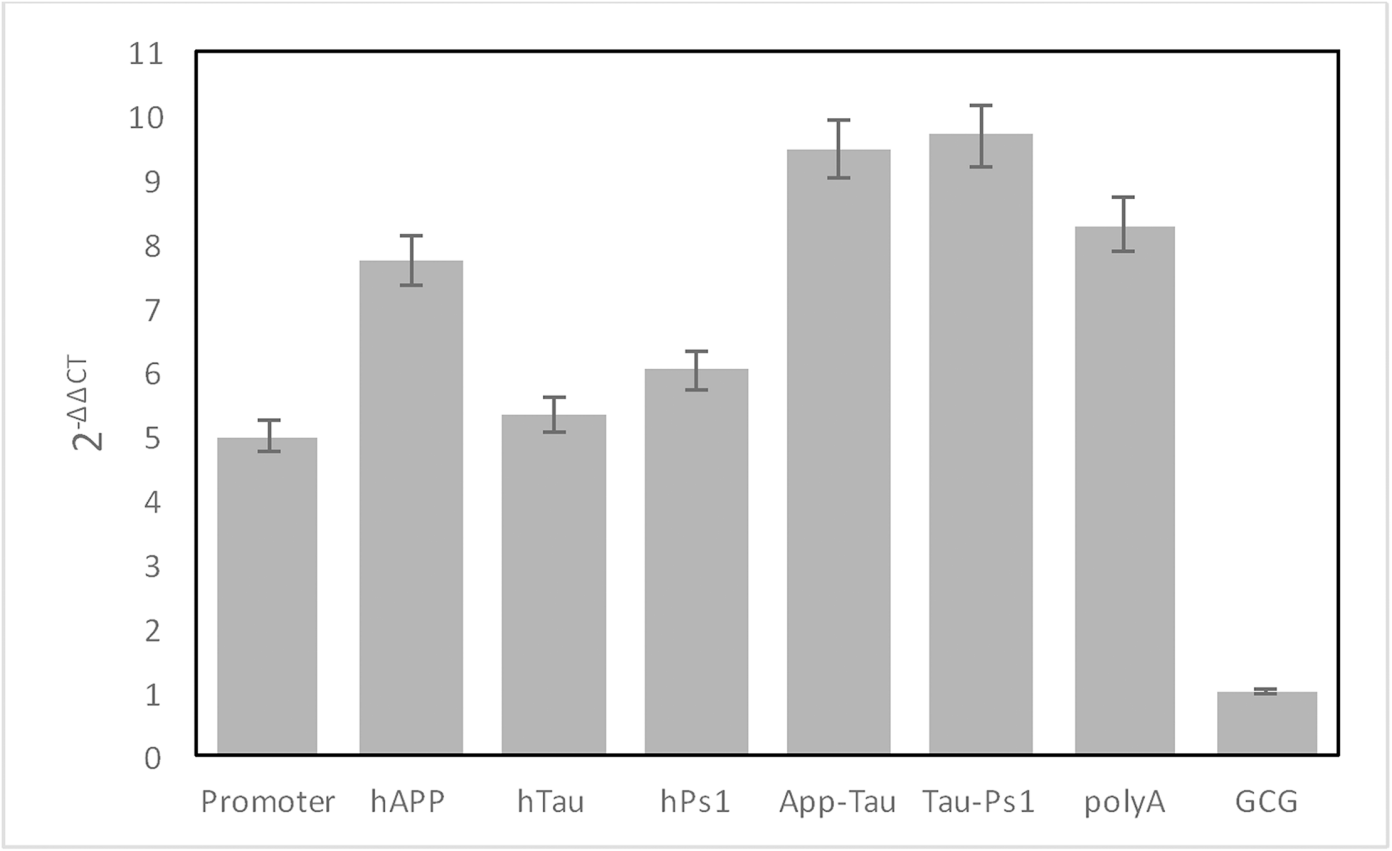
Estimation of copy number in the transgenic piglet genome. Real-time PCR was used to estimate the copy numbers of inserted transgenes. The X-axis indicates the inserted regions in the construct. An endogenous single-copy gene, glucagon (GCG), was used as a normalization control. Y-axis indicates 2^−ΔΔCT^ values. The results reveal that more than five copies of the inserts were present in the genome of the transgenic pig.

In another attempt to estimate transgene copy number, we carried out real-time quantitative PCR for each component of our Tg construct including the promoter, hAPP, hTau, hPS1, and poly A signals (Fig 7); the single-copy glucagon (GCG) gene was used as a normalization control. The results revealed that at least five copies of each Tg component were present in the genome of the Tg pig. These results are consistent with the results of the NGS analysis of gDNA from the Tg animal. However, in the case of the APP-Tau amplicons, the estimated copy numbers were much higher, suggesting the presence of nonfunctional partial integrations of the Tg construct elsewhere in the genome. These results are consistent with the observation of a large number of chimeric reads under less stringent selection conditions.

## Discussion

AD is a neurodegenerative disease, characterized pathologically by deposition of neurofibrillary tangles and amyloid plaques. This study sought to produce AD Tg pig by SCNT cloning using JB-PEF^AD^ cell lines. The parental cells were transfected with an AD multi-cistronic vector containing three mutant genes: APP, PS1, and Tau. SCNT with JB-PEF^AD^ (SCNT^AD^) embryos were transferred into the oviducts of surrogate mothers. After the cloned piglets were born, their genetic origin was confirmed by analysis of microsatellites, gDNA, mRNA, and protein, and transgene insertion sites and copy numbers were characterized. The transgenes were expressed at higher levels in brain than in other tissues. Furthermore, JNUPIG brain contained elevated levels of Aβ and activated astrocytes. The expression of triple AD mutant genes in Tg piglet could be finally developed AD. Thus, production of JNUPIGs using the SCNT technique is a feasible strategy for producing model pigs for research purposes.

To establish the AD Tg cell lines, JB-PEF cells were transfected using the AD multi-cistronic vector. Fibroblasts were chosen because they produce clones with high efficiency following SCNT [17]. Screening for Tg cells was necessary for production of Tg animals. Donor cell selection using antibiotic drugs often results in a mixed population of Tg and Non-Tg cells; consequently, the embryos derived from the donor cells are not always transgenic. In this study, we used hygromycin to select donor cells harboring the AD multi-cistronic vector, which contains a hygromycin resistance gene.

We performed SCNT using JB-PEF^AD^ as donor cells to identify the developmental potential of Tg embryos and confirm gDNA integration of the AD genes (hAPP, hTau, and hPS1) in pre-implantation embryos. The *in vitro* development potential of SCNT^AD^ embryos was slightly lower than that of SCNT^non-TG^ embryos, likely due to cell damage resulting from the transfection process (electric shock, insertion of viral vector, and antibiotic treatment) [18]. SCNT efficiencies are affected by various factors such as donor cell conditions, recipient oocyte quality, nuclear transfer technique, and genetic background. Blastocysts derived from SCNT^AD^ embryos successfully expressed the AD mutant genes (hAPP, hTau, and hPS1). Thus, blastocysts from SCNT^AD^ normally express the triple AD mutant genes derived from JB-PEF^AD^. From four tested AD Tg cell lines, we selected line #2–1 because it expressed the highest levels of the transgenes. Using this cell line, we obtained 30 SCNT^AD^ blastocysts with hAPP, hTau, and hPS1 genomic integrations, corresponding to 93.3% efficiency at the gDNA level.

ET and associated reproductive techniques enable the manipulation of germ cells and can, in conjunction with conventional breeding, improve the rate of genetic improvement of domestic animals [19]. To successfully clone TG piglets, it is necessary to consider age and estrus stage of surrogates, recovery of embryos, embryo handling, morphological assessment, intermediate storage, cultivation, transport, and transfer of recovered embryos into recipients [19]. The embryos, depending on the stage of development, are transferred in a small amount of medium using transfer pipettes or catheters, either into the oviduct (one- to four-cell embryos) or into the tip of the uterine horn (four-cell embryo to blastocyst). Embryos with a difference of one cell cycle on the stage of development can be accepted as transferable, whereas embryos in difference of more than two cell cycles have to be discharged. In this study, to produce the AD piglets, 1,834 SCNT embryos with JB-PEF^AD^ cells were transferred into surrogates, and two piglets with AD transgenes survived. The pregnancy and delivery rates may have been so low because the AD mutant genes impaired embryonic development. There are several difference between the use of aged beagles [20–23] and our AD causative gene transgenic pigs as AD animal models. Aged beagles can be considered as a disease model for sporadic AD comparing to AD gene transgenic pigs modelling inherited AD. In addition, AD transgenic pigs represent early onset inherited AD rather than sporadic AD in the dog model. The use of AD gene transgenic model may be more useful to investigate the specific mechanisms of AD because of the use of specific mutated genes causing AD in humans. The pig model is also more acceptable in production and ethical issues than the dog model. A previous study showed that PS1 plays an essential role in embryonic neurogenesis via Notch signaling [24]; in addition, PS1 has been implicated in several other biological pathways, including processing of APP, ErbB4, N- and E-cadherins, and CD44 [25], as well as the regulation of β-catenin stability [26]. To construct the multi-cistronic vector, porcine 2A sequences in psCMV vector were used. 2A system is one of well-known methods for expressing multiple genes. The system is based on “self-cleaving” in peptide sequences, not in DNA sequences like IRES elements. This system has been used for animal models and gene therapy in various diseases. Only problem with 2A system is an unwanted tag in a product due to cleavage occurring at the end of the 2A peptide sequence. This may affect the protein activity. Rather, it is not a problem in the 2A system, but most of the cloned animals die from cell replication in the early and middle stages of development. Even in the case of cloned animals that have been found to be alive, they are dying in infancy due to a variety of unknown diseases before they are fully matured. In our study, the healthy piglets had birth weights of 1.1 and 1.6 kg, within the range observed in our sow herds, and initial physical examinations revealed no abnormalities [27]. Thus, we successfully cloned JNUPIGs by SCNT using the AD multi-cistronic vector system.

We confirmed that the JNUPIGs expressed elevated levels of AD-related genes (APP, Tau and PS1) in multiple tissues. Moreover, even at very early time points, the AD Tg brain had high levels of Aβ in its brain, roughly 2-fold more than in controls. The ELISA kit for human Aβ40/42 is cross-reactive with porcine Aβ40/42, but nonetheless JNUPIG brain clearly contained more Aβ than control pig brain. We also monitored expression of Tau in JNUPIG brain using a total Tau ELISA kit, confirmed elevated total Tau level in JNUPIG brain. In addition, JNUPIG brain contained signs of astrocyte activation, a hallmark of AD-related inflammation.

NGS analysis of gDNA of the Tg animal revealed the structural characteristics of the inserted transgenes, and showed that the integration site was located within the intronic region between exons 9 and 10 of *BEND2*. Given that functional *BEND2* contains 12 exons in humans, the current site of transgene integration may disrupt the normal expression of the host gene. The function of *BEND2* is currently unknown, and it is expressed mainly in reproductive and immune tissues (GeneCards, http://www.genecards.org). Therefore, the effect of *BEND2* disruption must be considered in phenotypic analyses of transgene homozygotes. Also, the integration site of the transgene is on the X-chromosome, potentially resulting in reduced transgene expression due to X-chromosome inactivation. Future studies should further characterize the transgene integration sites and their functional consequences.

In summary, our results show that hAPP, hTau, and hPS1 were expressed efficiently from an AD multi-cistronic vector in transgenic porcine fibroblasts *in vitro*. The developmental ability of porcine Tg NT embryos derived from JB-PEF^AD^ cells was lower than that of non-Tg controls. The JNUPIGs generated using the multi-cistronic vector system harbored and expressed three AD-related mutant genes: human APP, Tau, and PS1. JNUPIGs could serve as valuable models for development and preclinical evaluation of neuro-based therapeutic strategies. Future investigations should seek to verify the onset of AD in these transgenic pigs, and then characterize the disorder at the cell, tissue, and organismal levels.

## Materials and methods

### Ethics statement

All animal studies was carried out in strict accordance with the recommendations in the Guide for the Care and Use Committee (IACUC) of the Jeju National University and the Guide for the Care and Use Committee (IACUC) of the National Institute of Animal Science. The Living Modified Organisms (LMO) was approved by Act on Transboundary Movement of Living Genetically Modified Organisms of Ministry of Science, ICT and Future Planning (No. LML16-128).

### Construction of AD using a multi-cistronic vector

The multi-cistronic vector was constructed using the retroviral vector pTet-CKOS (Fig 1A). A primer including a restriction enzyme site was manufactured and used to insert, into a vector, an hPDGF promoter, a CMV enhancer, and an APP gene (NM_201414.2) of a β-amyloid, a PS1 gene (NM_000021.3), a tau gene (NM_016834.4), and the like that cause the AD (Table 1). The human APP, PS1, and Tau genes were first cloned by PCR and then mutated using a site-directed mutagenesis kit (Stratagene, CA, USA). As an APP gene expressed in brain neurons was used, and triple mutations were introduced at amino acid positions 670 and 671, 716 and 717 in which familial mutations of the AD have been found. The mutations are known to form a larger amount of β-amyloid 42. The mutations are called "K670N/M617L", "I716V" and "V717I". In a tau gene, a single mutation occurs at amino acid position 301 and is called "P301L”. In a presenilin gene, two amino acid mutations were introduced. Mutations were introduced in amino acid positions 146 and 286 and are called "M146V" and "L286P" respectively. To separate three mutant genes as independent peptides when the three mutant genes are translated to proteins after transcription to a single messenger RNA (mRNA), the three mutant genes are connected to each other by 2A sequences. The vector includes mutated APP (NM_201414.2), PS1 (NM_000021.3), and Tau (NM_016834.4) genes, porcine 2A sequences and SV40 polyA-tail [28]. Human PDGFβ promoter were inserted in front of three mutant genes and CMV enhancer (CMVE) was also introduced upstream of the hPDGFβ to increase transgene expression in neurons [8]. The circular map of the final construct is shown in Fig 1B. The entire vector was confirmed by sequencing (S1 Fig). As an example of the recombinant expression vector, a base sequence of pTet-CMVE-hPDGFb-APPsw-2A-Tau-2A-PS1 may be defined with reference to SEQ ID NO: 9 described in the accompanying sequence list. The expression of the three AD-related genes was confirmed by transfection of the recombinant cell line into HEK293 (Fig 1C).

### Establishment of the AD cell line

Donor cell was originated from homotype-defined SLA (DQB1, DRB1, SLA1, SLA2, and SLA3) Jeju pigs (KNP, Subtropical Livestock Research Institute, NIAS, Jeju). For transfection, cells (1.0 × 10^6^) were suspended in 400 μL DPBS, 2.5 mg/mL AD multi-cistronic vector was added, and then electroporated under the following conditions: pulse voltage, 100 V; pulse width, 50 m; and pulse number, 2. Forty-eight hours after electroporation, the cells were transferred to DMEM culture medium containing 40 ng/mL hygromycin. At 12 days in selective culture, AD gene–positive cells (1.0 × 10^5^) were collected.

### Karyotyping

Cells were treated with 20 ng/mL colcemid (Irvine Scientific, USA) for 5 h, and then harvested. After treatment with 0.075 M KCl for 25 min at 37°C, cells were fixed by exposure to MeOH:acetic acid (3:1) (repeated three times), and the fixed cells were spread on slides. Chromosome images were captured using a Cytovision (Leica, Wetzlar, Germany).

### SCNT

The protocol was basically the same as the one described previously [29]. Pre-pubertal porcine ovaries were collected from a local slaughterhouse (Jeju Livestock Cooperative Products Marketing Center, Jeju, Republic of Korea) and transported to the laboratory in saline supplemented with 75 mg/ml penicillin G and 50 mg/ml streptomycin sulfate within 2 hours at 32~35°C. Cumulus-oocyte complexes (COCs) were aspirated from follicles 2 to 8 mm in diameter with an 18-gauge needle and a disposable 10 mL syringe. COCs were matured in TCM-199 (Gibco, Grand Island, NY, USA) containing Earle’s salts, cysteine, EGF, FSHLH, and 10% (v/v) porcine follicular fluid under mineral oil for 38 h at 38.8°C in 5% CO_2_ in air. For SCNT, cumulus cells were removed by gently pipetting in the presence of 1 mg/ mL hyaluronidase. The first polar body and nucleosome were enucleated on an Oosight imaging system (CRi, Hopkinton, MA, USA). The donor cell was inserted into the perivitelline space adjacent to cytoplasm. The karyoplast cytoplast complexes (KCC) were fused in fusion medium. Inserted donor cells were aligned to the northern wire in a fusion chamber (Lf201, Nepagene) with a direct current (DC) impulse of 110 V/cm for 60 μsec. The reconstructed embryos were activated with 7.5 μg/ml CB for 3 h. and then transferred to the PZM-5 medium supplemented with 0.4% FAF-BSA. On days 2 and 7, the cleavage and blastocyst formation rate was recorded based on the numbers of cleavages and blastocyst embryos.

### Surgical ET and pregnancy diagnosis

Crossbred prepubertal gilts (Large White/Landrace × Duroc) weighing 120–150 kg were used as recipients for the cloned embryos. The gilts from National Institute of Animal Science were raised at temperature of 23–25°C. Each of them was provided with a single stall in which they freely access water and ordinary feed. The gilts were given a single intramuscular injection of 1,000 IU equine chorionic gonadotropin (eCG, ASKA Pharmaceutical, Tokyo, Japan) to induce estrus. Ovulation was induced by intramuscular injection of 1,500 IU human chorionic gonadotropin (hCG, Kyoritsu Pharmaceutical, Tokyo, Japan) 66 h after the injection of eCG. The cloned embryos were cultured for 1 or 2 days, and then surgically transferred into the uterine horns of the recipients approximately 146 h after hCG injection. The SCNT embryos (≤150 embryos per surrogate) were transferred to the oviducts of naturally cycling gilts on the first day of standing estrus. Non-return surrogates were checked for pregnancy by transabdominal ultrasound examination on days 28 and 35 after ET. The experiment was repeated eleven independent times.

### PCR and real-time RT-PCR

gDNA was amplified in a 50 μL PCR reaction containing 5.0 units TaKaRa Ex Taq polymerase (Takara, Shiga, Japan), PCR amplification was carried out as follows: 1 cycle of denaturation at 94°C for 5 min; 40 cycles of denaturation at 94°C for 30 sec, annealing at 54°C for 30 sec, extension at 72°C for 30 sec; and a final extension at 72°C for 5 min. Aliquots (10 μL) of PCR products were fractionated on 0.8% or 1.2% agarose gels and stained with RedSafe™ Nucleic Acid Staining Solution (Intron, Seongnam, Korea).

Total RNA was isolated from brain, spleen, kidney, lung, heart, liver, and muscle using the TRIzol reagent, and real-time RT-PCR reactions were performed using the Quantitative Real-Time RT-PCR Analysis kit (Bio-Rad, Munich, Germany). RT-PCR involved an initial denaturation (95°Cfor 10 min), followed by 35 cycles of amplification and quantification (95°C for 10 sec, 55°Cfor 30 sec, and 72°C for 30 sec, with a single fluorescence measurement). The experiment was repeated three independent times. The primer sets for each gene are listed in Table 5.

**Table 5:** Primers used for PCR.

Purpose	Primer	GenBank accession no.	Primer sequence*	Annealing Temperature (°C)
Check of	GAPDH	AF017079	F:GGGCATGAACCATGAGAAGT	54
mRNA, gDNA			R:AAGCAGGGATGATGTTCTGG	
(cell, blastocyst)	hAPP	NM_201414.2	F:GAGTACCAACTTGCATGACTAC	54
			R:TCCTCCTCTGCTACTTCTACTAC	
	hTau	NM016834.4	F:GCTCAAAGGATAATATCAAACAC	54
			R:AAGTCAAGCTTCTCAGATTTTAC	
	hPS1	NM000021.3	F:TATATGATTTAGTGGCTGTTTTG	54
			R:TACTTGGAATTTTTGGATACTCT	
Integration	Promoter	-	F:GTGAGTACGTGTGACTGTGACTGAG	54
(cell, tissue)			R:GTCAGTCACCCTGCTGTTTACTATC	
	APP-2A-Tau	-	F:AACCTACAAGTTCTTTGAGCAGATG	54
			R:ATAGATCTAGGTCCAGGGTTCTCCT	
	Tau-2A-PS1	-	F:ATCTCAGCAATGTCTCCTCCAC	54
			R:ATTCTGGCTACGTACAGTATTGCTC	
	Poly A	-	F:GAGTTTGGACAAACCACAACTAGAA	54
			R:GCAAAAGCGAAACTACTATATCCTG	
	Poly A after vector	-	F:CTTGACGATTTTGACTTAGACATGC	54
			R:TAATCCAGAGGTTGATTAACAGGAA	
Real-time PCR	pACTB	-	F:GGACTTCGAGCAGGAGAT	54
(tissue)			R:GCACCGTGTTGGCGTAGAGG	
	APP-Tau	-	F:GAACGGCTACGAAAATCCAA	54
			R:CGTGTCACCCTCTTGGTCTT	
	Tau-PS1	-	F:ATCTCAGCAATGTCTCCTCCAC	54
			R:ATTCTGGCTACGTACAGTATTGCTC	

* F, forward; R, reverse.

### Western blot analysis

Cells were washed with PBS and lysed in RIPA buffer supplemented with a proteinase and phosphatase inhibitor cocktail (Sigma). Cell lysates were run on SDS-PAGE gels, and then transferred to PVDF membranes. After blocking with 5% (w/v) nonfat milk prepared in TBS for 1 h, the membrane was incubated for at least 2 h with primary antibody in TBST (20 mM Tris-HCl [pH 7.5], 250 mM NaCl, 0.05% (v/v) Tween®-20, and 5% (w/v) BSA), washed three times in TBST, and incubated for 1 h with anti-mouse and anti-rabbit IgG-HRP (Sigma & Santacruz) in TBST. After three washes with TBST, antibody binding was visualized with enhanced chemiluminescence (Amersham) followed by image analysis on a ChemiDoc (Bio-Rad). Primary antibodies were as follows: anti-hAPP (22C11; Chemicon & 6E10; Covance & 4G8; Biolegend), anti-hTau (Tau5; Invitrogen & H-150; Santacruz), anti-hPS1-CTF (Chemicon & H-70; Santacruz), anti-β-amyloid (Biolegend), anti-β-actin (Santacruz) and anti-GAPDH (Santacruz). The experiment was repeated three independent times.

### Microsatellite analysis

The tissue fragments and trypsinized donor cells were incubated overnight with analysis buffer (0.05 M Tris, 0.05 M EDTA, 0.5% SDS) supplemented with 400 g proteinase K, followed by phenol extraction and ethanol precipitation. The isolated gDNA samples were dissolved in 50 mL TE and used for microsatellite assays using 14 porcine DNA microsatellite markers (S02276, SW210, SW2410, S0107, S0277, S0068, SW2515, SW2401, SW1989, SWR1941, SWR1849, SW1337, S0018, and SW2456), each labeled with a fluorescent dye (FAM, TET, or HEX). Length variations were assayed on an automated DNA sequencer (ABI 373; Applied Biosystems, Foster City, CA, USA). Proprietary software (GeneScan and Genotyper; Applied Biosystems) was used to estimate PCR product size (in nucleotides).

### ELISA of biomarker levels in brain

Brains were homogenized in RIPA buffer using a sonicator (SONICS, USA) and serially diluted with ELISA sample buffer. Protein concentrations were determined using a BCA kit (Thermo Fisher Scientific). Quantitative measurements of Aβ1–40 and Aβ1–42 were performed using the Aβ1–40 and Aβ1–42 ELISA kits, respectively (IBL Japan). For each well, optical density at 450nm was measured on a Safire plate reader (Tecan, Switzerland), and Aβ1–40 and Aβ1–42 concentrations were determined by comparison with the corresponding standard curves. Total tau level was analyzed using total tau ELISA kit (Thermo Fisher Scientific) followed by user manual. The experiment was repeated three independent times.

### Immunohistochemistry

Brain was fixed in 10% neutral buffered formalin solution and cryopreserved in 30% sucrose/PBS. Brain was sectioned coronally on a cryostat (Leica, Germany) at 20 μm. Serial sections were collected in 0.1 M phosphate buffer, pH 7.6. Free-floating sections were incubated overnight at 4°C with primary antibody against the target protein. Primary antibody was detected using anti-human Aβ42 specific antibody (18582, IBL, 1:100) and anti-GFAP antibody (MAB3402, Millipore, 1:100). After several washes, the sections were incubated for 2 h at room temperature (RT) with goat anti-rabbit IgG antibody, Alexa Fluor 568 (A-11066, Molecular probe,1:200), goat anti-mouse IgG antibody, Alexa Fluor 488 (A-11054, Molecular probe,1:200). Nuclei were counterstained with 40, 6-diamidino-2-phenylindole (DAPI, Sigma–Aldrich). The experiment was repeated three independent times.

### Whole-genome sequencing

Isolated gDNA from an ear sample of the transgenic piglet was sequenced on a HiSeqX Ten system (Illumina, San Diego, CA, USA). The sequencing library was constructed using the TruSeq Nano DNA (350) kit (Illumina, San Diego, CA, USA). Briefly, the gDNA sample was randomly fragmented and ligated with adapter, and then adapter-ligated fragments were amplified and gel purified. Paired-end sequencing was performed with 150bp read length, and raw data were processed using the Real Time Analysis 2 (RTA 2) software (Illumina, San Diego, CA, USA). The base calls (BCL) binary was converted into FASTQ using bcl2fastq v2. 15.0 (Illumina, San Diego, CA, USA). Clean reads (over 60× depth) were mapped to the current pig genome assembly (Sscrofa 10.2) and to the sequence of transgenic construct using the Hisat 2 software [30] with default options.

### Insertion site identification

Chimeric reads containing sequences of both the pig genome and our construct were isolated for further analysis. Isolated reads were aligned using BLAST against the current pig genome assembly (Sscrofa 10.2) and our construct sequence to determine the insertion sites.

### Copy number identification

A single-copy endogenous control gene, GCG, was used to determine the copy number of the inserts in the transgenic piglet genome. PCR was performed on a Rotor-Gene Q System (Qiagen, Hilden, Germany) using the following amplification parameters: 5 min at 95°C; 40 cycles of 30 sec at 95°Cand 30 sec at 54°C, which generated a 130 bp amplicon. The primers used for the comprehensive amplification of inserts are listed in Table 5. The 2^-ΔΔCT^ method was used for the relative quantification of copy number [31].

### Statistics

All data were analyzed using a two-tailed Student’s t test. Data are expressed as the mean ± standard deviation (SD) or mean ± standard error of the mean (SEM).

## Supporting information

S1 Fig Sequence for AD multi-cistronic vector. (DOCX)

S1 Table The results of blast analysis on chimeric reads with high abundancy. (XLSX)
