# Supplementary material for: Production of transgenic pig as an Alzheimer’s disease model using a multi-cistronic vector system
Source: PLoS One. 2017 Jun 6;12(6):e0177933. doi: 10.1371/journal.pone.0177933 (PMC5460854; doi:10.1371/journal.pone.0177933)
Supplement: S1 Fig — (DOCX) [file pone.0177933.s001.docx]

**Supplementary Figure 1. Sequence for AD multi-cistronic vector**

**1. Modified Tet vector Sequence (7785 bp)**

tttgaaagaccccacccgtaggtggcaagctagcttaagtaacgccactttgcaaggcatggaaaaatacataactgagaatagaaaagttcagatcaaggtcaggaacaaagaaacagctgaataccaaacaggatatctgtggtaagcggttcctgccccggctcagggccaagaacagatgagacagctgagtgatgggccaaacaggatatctgtggtaagcagttcctgccccggctcggggccaagaacagatggtccccagatgcggtccagccctcagcagtttctagtgaatcatcagatgtttccagggtgccccaaggacctgaaaatgaccctgtaccttatttgaactaaccaatcagttcgcttctcgcttctgttcgcgcgcttccgctctccgagctcaataaaagagcccacaacccctcactcggcgcgccagtcttccgatagactgcgtcgcccgggtacccgtattcccaataaagcctcttgctgtttgcatccgaatcgtggtctcgctgttccttgggagggtctcctctgagtgattgactacccacgacgggggtctttcatttgggggctcgtccgggatttggagacccctgcccagggaccaccgacccaccaccgggaggtaagctggccagcaacttatctgtgtctgtccgattgtctagtgtctatgtttgatgttatgcgcctgcgtctgtactagttagctaactagctctgtatctggcggacccgtggtggaactgacgagttctgaacacccggccgcaaccctgggagacgtcccagggactttgggggccgtttttgtggcccgacctgaggaagggagtcgatgtggaatccgaccccgtcaggatatgtggttctggtaggagacgagaacctaaaacagttcccgcctccgtctgaatttttgctttcggtttggaaccgaagccgcgcgtcttgtctgctgcagcgctgcagcatcgttctgtgttgtctctgtctgactgtgtttctgtatttgtctgaaaattagggccagactgttaccactcccttaagtttgaccttaggtcactggaaagatgtcgagcggatcgctcacaaccagtcggtagatgtcaagaagagacgttgggttaccttctgctctgcagaatggccaacctttaacgtcggatggccgcgagacggcacctttaaccgagacctcatcacccaggttaagatcaaggtcttttcacctggcccgcatggacacccagaccaggtcccctacatcgtgacctgggaagccttggcttttgacccccctccctgggtcaagccctttgtacaccctaagcctccgcctcctcttcctccatccgccccgtctctcccccttgaacctcctcgttcgaccccgcctcgatcctccctttatccagccctcactccttctctaggcgccggaattccgatctgatagcttgccacaacccgtaccaaagatggatagatccggaaagcctgaactcaccgcgacgtctgtcgagaagtttctgatcgaaaagttcgacagcgtctccgacctgatgcagctctcggagggcgaagaatctcgtgctttcagcttcgatgtaggagggcgtggatatgtcctgcgggtaaatagctgcgccgatggtttctacaaagatcgttatgtttatcggcactttgcatcggccgcgctcccgattccggaagtgcttgacattggggaattcagcgagagcctgacctattgcatctcccgccgtgcacagggtgtcacgttgcaagacctgcctgaaaccgaactgcccgctgttctgcagccggtcgcggaggccatggatgcgatcgctgcggccgatcttagccagacgagcgggttcggcccattcggaccgcaaggaatcggtcaatacactacatggcgtgatttcatatgcgcgattgctgatccccatgtgtatcactggcaaactgtgatggacgacaccgtcagtgcgtccgtcgcgcaggctctcgatgagctgatgctttgggccgaggactgccccgaagtccggcacctcgtgcacgcggatttcggctccaacaatgtcctgacggacaatggccgcataacagcggtcattgactggagcgaggcgatgttcggggattcccaatacgaggtcgccaacatcttcttctggaggccgtggttggcttgtatggagcagcagacgcgctacttcgagcggaggcatccggagcttgcaggatcgccgcggctccgggcgtatatgctccgcattggtcttgaccaactctatcagagcttggttgacggcaatttcgatgatgcagcttgggcgcagggtcgatgcgacgcaatcgtccgatccggagccgggactgtcgggcgtacacaaatcgcccgcagaagcgcggccgtctggaccgatggctgtgtagaagtactcgccgatagtggaaaccgacgccccagcactcgtccgagggcaaaggaatagagtagatgccgaccgaacaagagctgatttcgagaacgcctcagccagcaactcgcgcgagcctagcaaggcaaatgcgagagaacggccttacgcttggtggcacagttctcgtccacagttcgctaagctcgctcggctgggtcgcgggagggccggtcgcagtgattcaggcccttctggattgtgttggtccccagggcacgattgtcatgcccacgcactcgggtgatctgactgatcccgcagattggagatcgccgcccgtgcctgccgattgggtgc**AGATCTATTTAAATACCGGTACGCGTATGCATTTAATTAAGTTTAAACATGCATCCTGCAGGGCGGCCGCCTCGAG**agatcccctcaggatatagtagtttcgcttttgcatagggagggggaaatgtagtcttatgcaatactcttgtagtcttgcaacatggtaacgatgagttagcaacatgccttacaaggagagaaaaagcaccgtgcatgccgattggtggaagtaaggtggtacgatcgtgccttattaggaaggcaacagacgggtctgacatggattggacgaaccactgaattccgcattgcagagatattgtatttaagtgcctagctcgatacagcaaacgccatttgaccattcaccacattggtgtgcacctccaagcttgttaattcaccatgtctagactggacaagagcaaagtcataaacggcgctctggaattactcaatggagtcggtatcgaaggcctgacgacaaggaaactcgctcaaaagctgggagttgagcagcctaccctgtactggcacgtgaagaacaagcgggccctgctcgatgccctgccaatcgagatgctggacaggcatcatacccacttctgccccctggaaggcgagtcatggcaagactttctgcggaacaacgccaagtcattccgctgtgctctcctctcacatcgcgacggggctaaagtgcatctcggcacccgcccaacagagaaacagtacgaaaccctggaaaatcagctcgcgttcctgtgtcagcaaggcttctccctggagaacgcactgtacgctctgtccgccgtgggccactttacactgggctgcgtattggaggaacaggagcatcaagtagcaaaagaggaaagagagacacctaccaccgattctatgcccccacttctgagacaagcaattgagctgttcgaccggcagggagccgaacctgccttccttttcggcctggaactaatcatatgtggcctggagaaacagctaaagtgcgaaagcggcgggccggccgacgcccttgacgattttgacttagacatgctcccagccgatgcccttgacgactttgaccttgatatgctgcctgctgacgctcttgacgattttgaccttgacatgctccccgggtaactaagtaaggatcaacatcgaattcgatttctgttcctgttaatcaacctctggattacaaaatttgtgaaagattgactggtattcttaactatgttgctccttttacgctatgtggatacgctgctttaatgcctttgtatcatgctattgcttcccgtatggctttcattttctcctccttgtataaatcctggttgctgtctctttatgaggagttgtggcccgttgtcaggcaacgtggcgtggtgtgcactgtgtttgctgacgcaacccccactggttggggcattgccaccacctgtcagctcctttccgggactttcgctttccccctccctattgccacggcggaactcatcgccgcctgccttgcccgctgctggacaggggctcggctgttgggcactgacaattccgtggtgttgtcggggaagctgacgtcctttccatggctgctcgcctgtgttgccacctggattctgcgcgggacgtccttctgctacgtcccttcggccctcaatccagcggaccttccttcccgcggcctgctgccggctctgcggcctcttccgcgtcttcgccttcgccctcagacgagtcggatctccctttgggccgcctccccgcctgtttcgcctcgggctcaatcactagtgaattcgataaaataaaagattttatttagtctccagaaaaaggggggaatgaaagaccccacctgtaggtttggcaagctagcttaagtaacgccattttgcaaggcatggaaaaatacataactgagaatagagaagttcagatcaaggtcaggaacagatggaacagctgaatatgggccaaacaggatatctgtggtaagcagttcctgccccggctcagggccaagaacagatggaacagctgaatatgggccaaacaggatatctgtggtaagcagttcctgccccggctcagggccaagaacagatggtccccagatgcggtccagccctcagcagtttctagagaaccatcagatgtttccagggtgccccaaggacctgaaatgaccctgtgccttatttgaactaaccaatcagttcgcttctcgcttctgttcgcgcgcttctgctccccgagctcaataaaagagcccacaacccctcactcggggcgccagtcctccgattgactgagtcgcccgggtacccgtgtatccaataaaccctcttgcagttgcatccgacttgtggtctcgctgttccttgggagggtctcctctgagtgattgactacccgtcagcgggggtctttcatttgggggctcgtccgggatcgggagacccctgcccagggaccaccgacccaccaccgggaggtaagctggctgcctcgcgcgtttcggtgatgacggtgaaaacctctgacacatgcagctcccggagacggtcacagcttgtctgtaagcggatgccgggagcagacaagcccgtcagggcgcgtcagcgggtgttggcgggtgtcggggcgcagccatgacccagtcacgtagcgatagcggagtgtatactggcttaactatgcggcatcagagcagattgtactgagagtgcaccatatgcggtgtgaaataccgcacagatgcgtaaggagaaaataccgcatcaggcgctcttccgcttcctcgctcactgactcgctgcgctcggtcgttcggctgcggcgagcggtatcagctcactcaaaggcggtaatacggttatccacagaatcaggggataacgcaggaaagaacatgtgagcaaaaggccagcaaaaggccaggaaccgtaaaaaggccgcgttgctggcgtttttccataggctccgcccccctgacgagcatcacaaaaatcgacgctcaagtcagaggtggcgaaacccgacaggactataaagataccaggcgtttccccctggaagctccctcgtgcgctctcctgttccgaccctgccgcttaccggatacctgtccgcctttctcccttcgggaagcgtggcgctttctcatagctcacgctgtaggtatctcagttcggtgtaggtcgttcgctccaagctgggctgtgtgcacgaaccccccgttcagcccgaccgctgcgccttatccggtaactatcgtcttgagtccaacccggtaagacacgacttatcgccactggcagcagccactggtaacaggattagcagagcgaggtatgtaggcggtgctacagagttcttgaagtggtggcctaactacggctacactagaaggacagtatttggtatctgcgctctgctgaagccagttaccttcggaaaaagagttggtagctcttgatccggcaaacaaaccaccgctggtagcggtggtttttttgtttgcaagcagcagattacgcgcagaaaaaaaggatctcaagaagatcctttgatcttttctacggggtctgacgctcagtggaacgaaaactcacgttaagggattttggtcatgagattatcaaaaaggatcttcacctagatccttttaaattaaaaatgaagttttaaatcaatctaaagtatatatgagtaaacttggtctgacagttaccaatgcttaatcagtgaggcacctatctcagcgatctgtctatttcgttcatccatagttgcctgactccccgtcgtgtagataactacgatacgggagggcttaccatctggccccagtgctgcaatgataccgcgagacccacgctcaccggctccagatttatcagcaataaaccagccagccggaagggccgagcgcagaagtggtcctgcaactttatccgcctccatccagtctattaattgttgccgggaagctagagtaagtagttcgccagttaatagtttgcgcaacgttgttgccattgctgcaggcatcgtggtgtcacgctcgtcgtttggtatggcttcattcagctccggttcccaacgatcaaggcgagttacatgatcccccatgttgtgcaaaaaagcggttagctccttcggtcctccgatcgttgtcagaagtaagttggccgcagtgttatcactcatggttatggcagcactgcataattctcttactgtcatgccatccgtaagatgcttttctgtgactggtgagtactcaaccaagtcattctgagaatagtgtatgcggcgaccgagttgctcttgcccggcgtcaacacgggataataccgcgccacatagcagaactttaaaagtgctcatcattggaaaacgttcttcggggcgaaaactctcaaggatcttaccgctgttgagatccagttcgatgtaacccactcgtgcacccaactgatcttcagcatcttttactttcaccagcgtttctgggtgagcaaaaacaggaaggcaaaatgccgcaaaaaagggaataagggcgacacggaaatgttgaatactcatactcttcctttttcaatattattgaagcatttatcagggttattgtctcatgagcggatacatatttgaatgtatttagaaaaataaacaaataggggttccgcgcacatttccccgaaaagtgccacctgacgtctaagaaaccattattatcatgacattaacctataaaaataggcgtatcacgaggccctttcgtcttcaagaattcataccagatcaccgaaaactgtcctccaaatgtgtccccctcacactcccaaattcgcgggcttctgcctcttagaccactctaccctattccccacactcaccggagccaaagccgcggcccttccgtttctttgct

**2. CMV enhancer (304 bp)**

CGTTACATAACTTACGGTAAATGGCCCGCCTGGCTGACCGCCCAACGACCCCCGCCCATTGACGTCAATAATGACGTATGTTCCCATAGTAACGCCAATAGGGACTTTCCATTGACGTCAATGGGTGGAGTATTTACGGTAAACTGCCCACTTGGCAGTACATCAAGTGTATCATATGCCAAGTACGCCCCCTATTGACGTCAATGACGGTAAATGGCCCGCCTGGCATTATGCCCAGTACATGACCTTATGGGACTTTCCTACTTGGCAGTACATCTACGTATTAGTCATCGCTATTACCATG

**3. human PDGFβ promoter sequence (1510 bp)**

GCTGGGACTACAGGAGCTTGTTACCACACCCAGCTCCAGTTTATAAATTCATCTCCAGTTTATAAAGGAGGAAACCGAGGTACTGAGAGGTTAAAAAACCTTCCTGCAGACACTTGTCCAGCAAGTGGCCACTCCAGGATTTGGACCAAGGTGATGTGTCTTCAGGCTGTGTCTCTGCCACTGTGCCACGCTGCTGGGTGGTAGGCAGCAGTGGGTGGGTGCCTGCAGTGGTCTGTAAAGACCACCTGAGATGTCCTTCCTCCTCTGTTCCACCCTGTCCAGGTCCAAGAAGACAGTCTATGAAGAGAGAGCAGGTGTGACTCTCTCAGTGTGCTCCTCTGTGAGAAGCAGGCTGACATCCCAAAGGGAAGGGCGGATAACAGAGACAGTGCAAGCGGAGGAGATGAGGGTGCCTCAAAGCCGGGAGGCTGGGTGATGCAGGAGCCTGCGTGTCCCGAGGGGGGTGCTGGGCCCAGTGTGAGTACGTGTGACTGTGACTGAGACAGTGTGACTGCTGAAGGCAGGGACACAGCAGCTCCCTGACTGGGGGCAGAAGGCGTTAACTGTGTGAAGGCTGGTTGTGGGTGGGTGGGCTCTGGGCCTCGAACCCGGGGGCTGAGGGAGATAGTAAACAGCAGGGTGACTGACGGGAAGATCATGTTGGTAGCCCTGCGAAGATGCTGCAGGGCTGTGGGGGTTTGTGTGACTTTGCAGTTCAACAAATTCAAATTCAGCCAACGCTGGCAGGGCCTGTTGTGCCAGGCAACCAGCTAGGAGGAGGAGACTCGGACCCAGCTTGCAGCTGAAGGGCGCTGGCTGCCGGGTTCTGTGGGTTCACCTTGCGGTGTCTTCCCTTGCTAACACTGAGTCCTTACAATAGCCCCATCTCCAGGTTGAGGCTAGATGGAGGGGACAGAGGGAAGTGACTTGCCCAAGGTGACCCAAGCTCCCGAGTGCCAGGGCAGGATCTGAATTCAGGCTCTCAGACTGCAGAGCCTGAGTCCCTCCCTGCCATGCCTGTGCCAGGGTGGAAATGTCTGGTCCTGGAGGGGAGCGTGGACTCCTGGCCTTGGCTCTGGAGACATCCCCCTAGACCACGTGGGCTCCTAACCTGTCCATGGTCACTGTGCTGAGGGGCGGGACGGTGGGTCACCCCTAGTTCTTTTTTCCCCAGGGCCAGATTCATGGACTGAAGGGTTGCTCGGCTCTCAGAGACCCCCTAAGCGCCCCGCCCTGGCCCCAAGCCCTCCCCCAGCTCCCGCGTCCCCCCCCTCCTGGCGCTGACTCCGGGCCAGAAGAGGAAAGGCTGTCTCCACCCACCTCTCGCACTCTCCCTTCTCCTTTATAAAGGCCGGAACAGCTGAAAGGGTGGCAACTTCTCCTCCTGCAGCCGGGAGCGGCCTGCCTGCCTCCCTGCGCACCCGCAGCCTCCCCCGCTGCCTCCCTAGGGCTCCCCTCCGGCCGCCAGCGCCCATTTTTCATTCCCTAGATAGAGATACTTTGCGCGCAC

**4. human APPsw sequence (2085 bp)**

**ATG**CTGCCCGGTTTGGCACTGCTCCTGCTGGCCGCCTGGACGGCTCGGGCGCTGGAGGTACCCACTGATGGTAATGCTGGCCTGCTGGCTGAACCCCAGATTGCCATGTTCTGTGGCAGACTGAACATGCACATGAATGTCCAGAATGGGAAGTGGGATTCAGATCCATCAGGGACCAAAACCTGCATTGATACCAAGGAAGGCATCCTGCAGTATTGCCAAGAAGTCTACCCTGAACTGCAGATCACCAATGTGGTAGAAGCCAACCAACCAGTGACCATCCAGAACTGGTGCAAGCGGGGCCGCAAGCAGTGCAAGACCCATCCCCACTTTGTGATTCCCTACCGCTGCTTAGTTGGTGAGTTTGTAAGTGATGCCCTTCTCGTTCCTGACAAGTGCAAATTCTTACACCAGGAGAGGATGGATGTTTGCGAAACTCATCTTCACTGGCACACCGTCGCCAAAGAGACATGCAGTGAGAAGAGTACCAACTTGCATGACTACGGCATGTTGCTGCCCTGCGGAATTGACAAGTTCCGAGGGGTAGAGTTTGTGTGTTGCCCACTGGCTGAAGAAAGTGACAATGTGGATTCTGCTGATGCGGAGGAGGATGACTCGGATGTCTGGTGGGGCGGAGCAGACACAGACTATGCAGATGGGAGTGAAGACAAAGTAGTAGAAGTAGCAGAGGAGGAAGAAGTGGCTGAGGTGGAAGAAGAAGAAGCCGATGATGACGAGGACGATGAGGATGGTGATGAGGTAGAGGAAGAGGCTGAGGAACCCTACGAAGAAGCCACAGAGAGAACCACCAGCATTGCCACCACCACCACCACCACCACAGAGTCTGTGGAAGAGGTGGTTCGAGTTCCTACAACAGCAGCCAGTACCCCTGATGCCGTTGACAAGTATCTCGAGACACCTGGGGATGAGAATGAACATGCCCATTTCCAGAAAGCCAAAGAGAGGCTTGAGGCCAAGCACCGAGAGAGAATGTCCCAGGTCATGAGAGAATGGGAAGAGGCAGAACGTCAAGCAAAGAACTTGCCTAAAGCTGATAAGAAGGCAGTTATCCAGCATTTCCAGGAGAAAGTGGAATCTTTGGAACAGGAAGCAGCCAACGAGAGACAGCAGCTGGTGGAGACACACATGGCCAGAGTGGAAGCCATGCTCAATGACCGCCGCCGCCTGGCCCTGGAGAACTACATCACCGCTCTGCAGGCTGTTCCTCCTCGGCCTCGTCACGTGTTCAATATGCTAAAGAAGTATGTCCGCGCAGAACAGAAGGACAGACAGCACACCCTAAAGCATTTCGAGCATGTGCGCATGGTGGATCCCAAGAAAGCCGCTCAGATCCGGTCCCAGGTTATGACACACCTCCGTGTGATTTATGAGCGCATGAATCAGTCTCTCTCCCTGCTCTACAACGTGCCTGCAGTGGCCGAGGAGATTCAGGATGAAGTTGATGAGCTGCTTCAGAAAGAGCAAAACTATTCAGATGACGTCTTGGCCAACATGATTAGTGAACCAAGGATCAGTTACGGAAACGATGCTCTCATGCCATCTTTGACCGAAACGAAAACCACCGTGGAGCTCCTTCCCGTGAATGGAGAGTTCAGCCTGGACGATCTCCAGCCGTGGCATTCTTTTGGGGCTGACTCTGTGCCAGCCAACACAGAAAACGAAGTTGAGCCTGTTGATGCCCGCCCTGCTGCCGACCGAGGACTGACCACTCGACCAGGTTCTGGGTTGACAAATATCAAGACGGAGGAGATCTCTGAAGTGAATCTGGATGCAGAATTCCGACATGACTCAGGATATGAAGTTCATCATCAAAAATTGGTGTTCTTTGCAGAAGATGTGGGTTCAAACAAAGGTGCAATCATTGGACTCATGGTGGGCGGTGTTGTCATAGCGACAGTGGTCATCATCACCTTGGTGATGCTGAAGAAGAAACAGTACACATCCATTCATCATGGTGTGGTGGAGGTTGACGCCGCTGTCACCCCAGAGGAGCGCCACCTGTCCAATCTGCAGCAGAACGGCTACGAAAATCCAACCTACAAGTTCTTTGAGCAGATGCAGAAC

**5. human Tau sequence (1149 bp)**

**ATG**GCTGAGCCCCGCCA**G**GAGTTCGAAGTGATGGAAGATCACGCTGGGACGTACGGGTTGGGGGACAGGAAAGATCAGGGGGGCTACACCATGCACCAAGACCAAGAGGGTGACACGGACGCTGGCCTGAAAGCTGAAGAAGCAGGCATTGGAGACACCCCCAGCCTGGAAGACGAAGCTGCTGGTCACGTGACCCAAGCTCGCATGGTCAGTAAAAGCAAAGACGGGACTGGAAGCGATGACAAAAAAGCCAAGGGGGCTGATGGTAAAACGAAGATCGCCACACCGCGGGGAGCAGCCCCTCCAGGCCAGAAGGGCCAGGCCAACGCCACCAGGATTCCAGCAAAAACCCCGCCCGCTCCAAAGACACCACCCAGCTCTGGTGAACCTCCAAAATCAGGGGATCGCAGCGGCTACAGCAGCCCCGGCTCCCCAGGCACTCCCGGCAGCCGCTCCCGCACCCCGTCCCTTCCAACCCCACCCACCCGGGAGCCCAAGAAGGTGGCAGTGGTCCGTACTCCACCCAAGTCGCCGTCTTCCGCCAAGAGCCGCCTGCAGACAGCCCCCGTGCCCATGCCAGACCTGAAGAATGTCAAGTCCAAGATCGGCTCCACTGAGAACCTGAAGCACCAGCCGGGAGGCGGGAAGGTGCAGATAATTAATAAGAAGCTGGATCTTAGCAACGTCCAGTCCAAGTGTGGCTCAAAGGATAATATCAAACACGTCCTGGGAGGCGGCAGTGTGCAAATAGTCTACAAACCAGTTGACCTGAGCAAGGTGACCTCCAAGTGTGGCTCATTAGGCAACATCCATCATAAACCAGGAGGTGGCCAGGTGGAAGTAAAATCTGAGAAGCTTGACTTCAAGGACAGAGTCCAGTCGAAGATTGGGTCCCTGGACAATATCACCCACGTCCCTGGCGGAGGAAATAAAAAGATTGAAACCCACAAGCTGACCTTCCGCGAGAACGCCAAAGCCAAGACAGACCACGGGGCGGAGATCGTGTACAAGTCGCCAGTGGTGTCTGGGGACACGTCTCCACGGCATCTCAGCAATGTCTCCTCCACCGGCAGCATCGACATGGTAGACTCGCCCCAGCTCGCCACGCTAGCTGACGAGGTGTCTGCCTCCCTGGCCAAGCAGGGTTTG

**6. human PS1 sequence (1404 bp)**

**ATG**ACAGAGTTACCTGCACCGTTGTCCTACTTCCAGAATGCACAGATGTCTGAGGACAACCACCTGAGCAATACTGTACGTAGCCAGAATGACAATAGAGAACGGCAGGAGCACAACGACAGACGGAGCCTTGGCCACCCTGAGCCATTATCTAATGGACGACCCCAGGGTAACTCCCGGCAGGTGGTGGAGCAAGATGAGGAAGAAGATGAGGAGCTGACATTGAAATATGGCGCCAAGCATGTGATCATGCTCTTTGTCCCTGTGACTCTCTGCATGGTGGTGGTCGTGGCTACCATTAAGTCAGTCAGCTTTTATACCCGGAAGGATGGGCAGCTAATCTATACCCCATTCACAGAAGATACCGAGACTGTGGGCCAGAGAGCCCTGCACTCAATTCTGAATGCTGCCATCATGATCAGTGTCATTGTTGTCCTGACTATCCTCCTGGTGGTTCTGTATAAATACAGGTGCTATAAGGTCATCCATGCCTGGCTTATTATATCATCTCTATTGTTGCTGTTCTTTTTTTCATTCATTTACTTGGGGGAAGTGTTTAAAACCTATAACGTTGCTGTGGACTACATTACTGTTGCACTCCTGATCTGGAATTTTGGTGTGGTGGGAATGATTTCCATTCACTGGAAAGGTCCACTTCGACTCCAGCAGGCATATCTCATTATGATTAGTGCCCTCATGGCCCTGGTGTTTATCAAGTACCTCCCTGAATGGACTGCGTGGCTCATCTTGGCTGTGATTTCAGTATATGATTTAGTGGCTGTTTTGTGTCCGAAAGGTCCACTTCGTATGCTGGTTGAAACAGCTCAGGAGAGAAATGAAACGCTTTTTCCAGCTGTCATTTACTCCTCAACAATGGTGTGGTTGGTGAATATGGCAGAAGGAGACCCGGAAGCTCAAAGGAGAGTATCCAAAAATTCCAAGTATAATGCAGAAAGCACAGAAAGGGAGTCACAAGACACTGTTGCAGAGAATGATGATGGCGGGTTCAGTGAGGAATGGGAAGCCCAGAGGGACAGTCATCTAGGGCCTCATCGCTCTACACCTGAGTCACGAGCTGCTGTCCAGGAACTTTCCAGCAGTATCCTCGCTGGTGAAGACCCAGAGGAAAGGGGAGTAAAACTTGGATTGGGAGATTTCATTTTCTACAGTGTTCTGGTTGGTAAAGCCTCAGCAACAGCCAGTGGAGACTGGAACACAACCATAGCCTGTTTCGTAGCCATATTAATTGGTTTGTGCCTTACATTATTACTCCTTGCCATTTTCAAGAAAGCATTGCCAGCTCTTCCAATCTCCATCACCTTTGGGCTTGTTTTCTACTTTGCCACAGATTATCTTGTACAGCCTTTTATGGACCAATTAGCATTCCATCAATTTTATATC**TAG**

**7. SV40 poly A sequence (186 bp)**

AGATACATTGATGAGTTTGGACAAACCACAACTAGAATGCAGTGAAAAAAATGCTTTATTTGTGAAATTTGTGATGCTATTGCTTTATTTGTAACCATTATAAGCTGCAATAAACAAGTTAACAACAACAATTGCATTCATTTTATGTTTCAGGTTCAGGGGGAGGTGTGGGAGGTTTTTTAATTC

**8. 2A sequence**

GGAAGCGGAGCTACTAACTTCAGCCTGCTGAAGCAGGCTGGAGACGTGGAGGAGAACCCTGGACCT

**9. final construct pTet-CMVE-hPDGFb-APPsw-2A-Tau-2A-PS1 (14,305 bp)**

tttgaaagaccccacccgtaggtggcaagctagcttaagtaacgccactttgcaaggcatggaaaaatacataactgagaatagaaaagttcagatcaaggtcaggaacaaagaaacagctgaataccaaacaggatatctgtggtaagcggttcctgccccggctcagggccaagaacagatgagacagctgagtgatgggccaaacaggatatctgtggtaagcagttcctgccccggctcggggccaagaacagatggtccccagatgcggtccagccctcagcagtttctagtgaatcatcagatgtttccagggtgccccaaggacctgaaaatgaccctgtaccttatttgaactaaccaatcagttcgcttctcgcttctgttcgcgcgcttccgctctccgagctcaataaaagagcccacaacccctcactcggcgcgccagtcttccgatagactgcgtcgcccgggtacccgtattcccaataaagcctcttgctgtttgcatccgaatcgtggtctcgctgttccttgggagggtctcctctgagtgattgactacccacgacgggggtctttcatttgggggctcgtccgggatttggagacccctgcccagggaccaccgacccaccaccgggaggtaagctggccagcaacttatctgtgtctgtccgattgtctagtgtctatgtttgatgttatgcgcctgcgtctgtactagttagctaactagctctgtatctggcggacccgtggtggaactgacgagttctgaacacccggccgcaaccctgggagacgtcccagggactttgggggccgtttttgtggcccgacctgaggaagggagtcgatgtggaatccgaccccgtcaggatatgtggttctggtaggagacgagaacctaaaacagttcccgcctccgtctgaatttttgctttcggtttggaaccgaagccgcgcgtcttgtctgctgcagcgctgcagcatcgttctgtgttgtctctgtctgactgtgtttctgtatttgtctgaaaattagggccagactgttaccactcccttaagtttgaccttaggtcactggaaagatgtcgagcggatcgctcacaaccagtcggtagatgtcaagaagagacgttgggttaccttctgctctgcagaatggccaacctttaacgtcggatggccgcgagacggcacctttaaccgagacctcatcacccaggttaagatcaaggtcttttcacctggcccgcatggacacccagaccaggtcccctacatcgtgacctgggaagccttggcttttgacccccctccctgggtcaagccctttgtacaccctaagcctccgcctcctcttcctccatccgccccgtctctcccccttgaacctcctcgttcgaccccgcctcgatcctccctttatccagccctcactccttctctaggcgccggaattccgatctgatagcttgccacaacccgtaccaaagatggatagatccggaaagcctgaactcaccgcgacgtctgtcgagaagtttctgatcgaaaagttcgacagcgtctccgacctgatgcagctctcggagggcgaagaatctcgtgctttcagcttcgatgtaggagggcgtggatatgtcctgcgggtaaatagctgcgccgatggtttctacaaagatcgttatgtttatcggcactttgcatcggccgcgctcccgattccggaagtgcttgacattggggaattcagcgagagcctgacctattgcatctcccgccgtgcacagggtgtcacgttgcaagacctgcctgaaaccgaactgcccgctgttctgcagccggtcgcggaggccatggatgcgatcgctgcggccgatcttagccagacgagcgggttcggcccattcggaccgcaaggaatcggtcaatacactacatggcgtgatttcatatgcgcgattgctgatccccatgtgtatcactggcaaactgtgatggacgacaccgtcagtgcgtccgtcgcgcaggctctcgatgagctgatgctttgggccgaggactgccccgaagtccggcacctcgtgcacgcggatttcggctccaacaatgtcctgacggacaatggccgcataacagcggtcattgactggagcgaggcgatgttcggggattcccaatacgaggtcgccaacatcttcttctggaggccgtggttggcttgtatggagcagcagacgcgctacttcgagcggaggcatccggagcttgcaggatcgccgcggctccgggcgtatatgctccgcattggtcttgaccaactctatcagagcttggttgacggcaatttcgatgatgcagcttgggcgcagggtcgatgcgacgcaatcgtccgatccggagccgggactgtcgggcgtacacaaatcgcccgcagaagcgcggccgtctggaccgatggctgtgtagaagtactcgccgatagtggaaaccgacgccccagcactcgtccgagggcaaaggaatagagtagatgccgaccgaacaagagctgatttcgagaacgcctcagccagcaactcgcgcgagcctagcaaggcaaatgcgagagaacggccttacgcttggtggcacagttctcgtccacagttcgctaagctcgctcggctgggtcgcgggagggccggtcgcagtgattcaggcccttctggattgtgttggtccccagggcacgattgtcatgcccacgcactcgggtgatctgactgatcccgcagattggagatcgccgcccgtgcctgccgattgggtgcaGATCTATTTAAATGCGTTACATAACTTACGGTAAATGGCCCGCCTGGCTGACCGCCCAACGACCCCCGCCCATTGACGTCAATAATGACGTATGTTCCCATAGTAACGCCAATAGGGACTTTCCATTGACGTCAATGGGTGGAGTATTTACGGTAAACTGCCCACTTGGCAGTACATCAAGTGTATCATATGCCAAGTACGCCCCCTATTGACGTCAATGACGGTAAATGGCCCGCCTGGCATTATGCCCAGTACATGACCTTATGGGACTTTCCTACTTGGCAGTACATCTACGTATTAGTCATCGCTATTACCATGATTTAAATCTGGGACTACAGGAGCTTGTTACCACACCCAGCTCCAGTTTATAAATTCATCTCCAGTTTATAAAGGAGGAAACCGAGGTACTGAGAGGTTAAAAAACCTTCCTGCAGACACTTGTCCAGCAAGTGGCCACTCCAGGATTTGGACCAAGGTGATGTGTCTTCAGGCTGTGTCTCTGCCACTGTGCCACGCTGCTGGGTGGTAGGCAGCAGTGGGTGGGTGCCTGCAGTGGTCTGTAAAGACCACCTGAGATGTCCTTCCTCCTCTGTTCCACCCTGTCCAGGTCCAAGAAGACAGTCTATGAAGAGAGAGCAGGTGTGACTCTCTCAGTGTGCTCCTCTGTGAGAAGCAGGCTGACATCCCAAAGGGAAGGGCGGATAACAGAGACAGTGCAAGCGGAGGAGATGAGGGTGCCTCAAAGCCGGGAGGCTGGGTGATGCAGGAGCCTGCGTGTCCCGAGGGGGGTGCTGGGCCCAGTGTGAGTACGTGTGACTGTGACTGAGACAGTGTGACTGCTGAAGGCAGGGACACAGCAGCTCCCTGACTGGGGGCAGAAGGCGTTAACTGTGTGAAGGCTGGTTGTGGGTGGGTGGGCTCTGGGCCTCGAACCCGGGGGCTGAGGGAGATAGTAAACAGCAGGGTGACTGACGGGAAGATCATGTTGGTAGCCCTGCGAAGATGCTGCAGGGCTGTGGGGGTTTGTGTGACTTTGCAGTTCAACAAATTCAAATTCAGCCAACGCTGGCAGGGCCTGTTGTGCCAGGCAACCAGCTAGGAGGAGGAGACTCGGACCCAGCTTGCAGCTGAAGGGCGCTGGCTGCCGGGTTCTGTGGGTTCACCTTGCGGTGTCTTCCCTTGCTAACACTGAGTCCTTACAATAGCCCCATCTCCAGGTTGAGGCTAGATGGAGGGGACAGAGGGAAGTGACTTGCCCAAGGTGACCCAAGCTCCCGAGTGCCAGGGCAGGATCTGAATTCAGGCTCTCAGACTGCAGAGCCTGAGTCCCTCCCTGCCATGCCTGTGCCAGGGTGGAAATGTCTGGTCCTGGAGGGGAGCGTGGACTCCTGGCCTTGGCTCTGGAGACATCCCCCTAGACCACGTGGGCTCCTAACCTGTCCATGGTCACTGTGCTGAGGGGCGGGACGGTGGGTCACCCCTAGTTCTTTTTTCCCCAGGGCCAGATTCATGGACTGAAGGGTTGCTCGGCTCTCAGAGACCCCCTAAGCGCCCCGCCCTGGCCCCAAGCCCTCCCCCAGCTCCCGCGTCCCCCCCCTCCTGGCGCTGACTCCGGGCCAGAAGAGGAAAGGCTGTCTCCACCCACCTCTCGCACTCTCCCTTCTCCTTTATAAAGGCCGGAACAGCTGAAAGGGTGGCAACTTCTCCTCCTGCAGCCGGGAGCGGCCTGCCTGCCTCCCTGCGCACCCGCAGCCTCCCCCGCTGCCTCCCTAGGGCTCCCCTCCGGCCGCCAGCGCCCATTTTTCATTCCCTAGATAGAGATACTTTGCGCGCACatcgat**ATG**CTGCCCGGTTTGGCACTGCTCCTGCTGGCCGCCTGGACGGCTCGGGCGCTGGAGGTACCCACTGATGGTAATGCTGGCCTGCTGGCTGAACCCCAGATTGCCATGTTCTGTGGCAGACTGAACATGCACATGAATGTCCAGAATGGGAAGTGGGATTCAGATCCATCAGGGACCAAAACCTGCATTGATACCAAGGAAGGCATCCTGCAGTATTGCCAAGAAGTCTACCCTGAACTGCAGATCACCAATGTGGTAGAAGCCAACCAACCAGTGACCATCCAGAACTGGTGCAAGCGGGGCCGCAAGCAGTGCAAGACCCATCCCCACTTTGTGATTCCCTACCGCTGCTTAGTTGGTGAGTTTGTAAGTGATGCCCTTCTCGTTCCTGACAAGTGCAAATTCTTACACCAGGAGAGGATGGATGTTTGCGAAACTCATCTTCACTGGCACACCGTCGCCAAAGAGACATGCAGTGAGAAGAGTACCAACTTGCATGACTACGGCATGTTGCTGCCCTGCGGAATTGACAAGTTCCGAGGGGTAGAGTTTGTGTGTTGCCCACTGGCTGAAGAAAGTGACAATGTGGATTCTGCTGATGCGGAGGAGGATGACTCGGATGTCTGGTGGGGCGGAGCAGACACAGACTATGCAGATGGGAGTGAAGACAAAGTAGTAGAAGTAGCAGAGGAGGAAGAAGTGGCTGAGGTGGAAGAAGAAGAAGCCGATGATGACGAGGACGATGAGGATGGTGATGAGGTAGAGGAAGAGGCTGAGGAACCCTACGAAGAAGCCACAGAGAGAACCACCAGCATTGCCACCACCACCACCACCACCACAGAGTCTGTGGAAGAGGTGGTTCGAGTTCCTACAACAGCAGCCAGTACCCCTGATGCCGTTGACAAGTATCTCGAGACACCTGGGGATGAGAATGAACATGCCCATTTCCAGAAAGCCAAAGAGAGGCTTGAGGCCAAGCACCGAGAGAGAATGTCCCAGGTCATGAGAGAATGGGAAGAGGCAGAACGTCAAGCAAAGAACTTGCCTAAAGCTGATAAGAAGGCAGTTATCCAGCATTTCCAGGAGAAAGTGGAATCTTTGGAACAGGAAGCAGCCAACGAGAGACAGCAGCTGGTGGAGACACACATGGCCAGAGTGGAAGCCATGCTCAATGACCGCCGCCGCCTGGCCCTGGAGAACTACATCACCGCTCTGCAGGCTGTTCCTCCTCGGCCTCGTCACGTGTTCAATATGCTAAAGAAGTATGTCCGCGCAGAACAGAAGGACAGACAGCACACCCTAAAGCATTTCGAGCATGTGCGCATGGTGGATCCCAAGAAAGCCGCTCAGATCCGGTCCCAGGTTATGACACACCTCCGTGTGATTTATGAGCGCATGAATCAGTCTCTCTCCCTGCTCTACAACGTGCCTGCAGTGGCCGAGGAGATTCAGGATGAAGTTGATGAGCTGCTTCAGAAAGAGCAAAACTATTCAGATGACGTCTTGGCCAACATGATTAGTGAACCAAGGATCAGTTACGGAAACGATGCTCTCATGCCATCTTTGACCGAAACGAAAACCACCGTGGAGCTCCTTCCCGTGAATGGAGAGTTCAGCCTGGACGATCTCCAGCCGTGGCATTCTTTTGGGGCTGACTCTGTGCCAGCCAACACAGAAAACGAAGTTGAGCCTGTTGATGCCCGCCCTGCTGCCGACCGAGGACTGACCACTCGACCAGGTTCTGGGTTGACAAATATCAAGACGGAGGAGATCTCTGAAGTGAATCTGGATGCAGAATTCCGACATGACTCAGGATATGAAGTTCATCATCAAAAATTGGTGTTCTTTGCAGAAGATGTGGGTTCAAACAAAGGTGCAATCATTGGACTCATGGTGGGCGGTGTTGTCATAGCGACAGTGGTCATCATCACCTTGGTGATGCTGAAGAAGAAACAGTACACATCCATTCATCATGGTGTGGTGGAGGTTGACGCCGCTGTCACCCCAGAGGAGCGCCACCTGTCCAATCTGCAGCAGAACGGCTACGAAAATCCAACCTACAAGTTCTTTGAGCAGATGCAGAACttaattaaggcatgcGGAAGCGGAGCTACTAACTTCAGCCTGCTGAAGCAGGCTGGAGACGTGGAGGAGAACCCTGGACCTagatct**ATG**GCTGAGCCCCGCCA**G**GAGTTCGAAGTGATGGAAGATCACGCTGGGACGTACGGGTTGGGGGACAGGAAAGATCAGGGGGGCTACACCATGCACCAAGACCAAGAGGGTGACACGGACGCTGGCCTGAAAGCTGAAGAAGCAGGCATTGGAGACACCCCCAGCCTGGAAGACGAAGCTGCTGGTCACGTGACCCAAGCTCGCATGGTCAGTAAAAGCAAAGACGGGACTGGAAGCGATGACAAAAAAGCCAAGGGGGCTGATGGTAAAACGAAGATCGCCACACCGCGGGGAGCAGCCCCTCCAGGCCAGAAGGGCCAGGCCAACGCCACCAGGATTCCAGCAAAAACCCCGCCCGCTCCAAAGACACCACCCAGCTCTGGTGAACCTCCAAAATCAGGGGATCGCAGCGGCTACAGCAGCCCCGGCTCCCCAGGCACTCCCGGCAGCCGCTCCCGCACCCCGTCCCTTCCAACCCCACCCACCCGGGAGCCCAAGAAGGTGGCAGTGGTCCGTACTCCACCCAAGTCGCCGTCTTCCGCCAAGAGCCGCCTGCAGACAGCCCCCGTGCCCATGCCAGACCTGAAGAATGTCAAGTCCAAGATCGGCTCCACTGAGAACCTGAAGCACCAGCCGGGAGGCGGGAAGGTGCAGATAATTAATAAGAAGCTGGATCTTAGCAACGTCCAGTCCAAGTGTGGCTCAAAGGATAATATCAAACACGTCCTGGGAGGCGGCAGTGTGCAAATAGTCTACAAACCAGTTGACCTGAGCAAGGTGACCTCCAAGTGTGGCTCATTAGGCAACATCCATCATAAACCAGGAGGTGGCCAGGTGGAAGTAAAATCTGAGAAGCTTGACTTCAAGGACAGAGTCCAGTCGAAGATTGGGTCCCTGGACAATATCACCCACGTCCCTGGCGGAGGAAATAAAAAGATTGAAACCCACAAGCTGACCTTCCGCGAGAACGCCAAAGCCAAGACAGACCACGGGGCGGAGATCGTGTACAAGTCGCCAGTGGTGTCTGGGGACACGTCTCCACGGCATCTCAGCAATGTCTCCTCCACCGGCAGCATCGACATGGTAGACTCGCCCCAGCTCGCCACGCTAGCTGACGAGGTGTCTGCCTCCCTGGCCAAGCAGGGTTTGGAATTCGGAAGCGGAGCTACTAACTTCAGCCTGCTGAAGCAGGCTGGAGACGTGGAGGAGAACCCTGGACCTCTCGAG**ATG**ACAGAGTTACCTGCACCGTTGTCCTACTTCCAGAATGCACAGATGTCTGAGGACAACCACCTGAGCAATACTGTACGTAGCCAGAATGACAATAGAGAACGGCAGGAGCACAACGACAGACGGAGCCTTGGCCACCCTGAGCCATTATCTAATGGACGACCCCAGGGTAACTCCCGGCAGGTGGTGGAGCAAGATGAGGAAGAAGATGAGGAGCTGACATTGAAATATGGCGCCAAGCATGTGATCATGCTCTTTGTCCCTGTGACTCTCTGCATGGTGGTGGTCGTGGCTACCATTAAGTCAGTCAGCTTTTATACCCGGAAGGATGGGCAGCTAATCTATACCCCATTCACAGAAGATACCGAGACTGTGGGCCAGAGAGCCCTGCACTCAATTCTGAATGCTGCCATCATGATCAGTGTCATTGTTGTCCTGACTATCCTCCTGGTGGTTCTGTATAAATACAGGTGCTATAAGGTCATCCATGCCTGGCTTATTATATCATCTCTATTGTTGCTGTTCTTTTTTTCATTCATTTACTTGGGGGAAGTGTTTAAAACCTATAACGTTGCTGTGGACTACATTACTGTTGCACTCCTGATCTGGAATTTTGGTGTGGTGGGAATGATTTCCATTCACTGGAAAGGTCCACTTCGACTCCAGCAGGCATATCTCATTATGATTAGTGCCCTCATGGCCCTGGTGTTTATCAAGTACCTCCCTGAATGGACTGCGTGGCTCATCTTGGCTGTGATTTCAGTATATGATTTAGTGGCTGTTTTGTGTCCGAAAGGTCCACTTCGTATGCTGGTTGAAACAGCTCAGGAGAGAAATGAAACGCTTTTTCCAGCTGTCATTTACTCCTCAACAATGGTGTGGTTGGTGAATATGGCAGAAGGAGACCCGGAAGCTCAAAGGAGAGTATCCAAAAATTCCAAGTATAATGCAGAAAGCACAGAAAGGGAGTCACAAGACACTGTTGCAGAGAATGATGATGGCGGGTTCAGTGAGGAATGGGAAGCCCAGAGGGACAGTCATCTAGGGCCTCATCGCTCTACACCTGAGTCACGAGCTGCTGTCCAGGAACTTTCCAGCAGTATCCTCGCTGGTGAAGACCCAGAGGAAAGGGGAGTAAAACTTGGATTGGGAGATTTCATTTTCTACAGTGTTCTGGTTGGTAAAGCCTCAGCAACAGCCAGTGGAGACTGGAACACAACCATAGCCTGTTTCGTAGCCATATTAATTGGTTTGTGCCTTACATTATTACTCCTTGCCATTTTCAAGAAAGCATTGCCAGCTCTTCCAATCTCCATCACCTTTGGGCTTGTTTTCTACTTTGCCACAGATTATCTTGTACAGCCTTTTATGGACCAATTAGCATTCCATCAATTTTATATC**TAG**cctgcaggTCTAGATAGCTAGCCTCCCTATAGTGAGTCGTATTACGTAGATCCAGACATGATAAGATACATTGATGAGTTTGGACAAACCACAACTAGAATGCAGTGAAAAAAATGCTTTATTTGTGAAATTTGTGATGCTATTGCTTTATTTGTAACCATTATAAGCTGCAATAAACAAGTTAACAACAACAATTGCATTCATTTTATGTTTCAGGTTCAGGGGGAGGTGTGGGAGGTTTTTTAATTCGCGGCCGCCTCGAGagatcccctcaggatatagtagtttcgcttttgcatagggagggggaaatgtagtcttatgcaatactcttgtagtcttgcaacatggtaacgatgagttagcaacatgccttacaaggagagaaaaagcaccgtgcatgccgattggtggaagtaaggtggtacgatcgtgccttattaggaaggcaacagacgggtctgacatggattggacgaaccactgaattccgcattgcagagatattgtatttaagtgcctagctcgatacagcaaacgccatttgaccattcaccacattggtgtgcacctccaagcttgttaattcaccatgtctagactggacaagagcaaagtcataaacggcgctctggaattactcaatggagtcggtatcgaaggcctgacgacaaggaaactcgctcaaaagctgggagttgagcagcctaccctgtactggcacgtgaagaacaagcgggccctgctcgatgccctgccaatcgagatgctggacaggcatcatacccacttctgccccctggaaggcgagtcatggcaagactttctgcggaacaacgccaagtcattccgctgtgctctcctctcacatcgcgacggggctaaagtgcatctcggcacccgcccaacagagaaacagtacgaaaccctggaaaatcagctcgcgttcctgtgtcagcaaggcttctccctggagaacgcactgtacgctctgtccgccgtgggccactttacactgggctgcgtattggaggaacaggagcatcaagtagcaaaagaggaaagagagacacctaccaccgattctatgcccccacttctgagacaagcaattgagctgttcgaccggcagggagccgaacctgccttccttttcggcctggaactaatcatatgtggcctggagaaacagctaaagtgcgaaagcggcgggccggccgacgcccttgacgattttgacttagacatgctcccagccgatgcccttgacgactttgaccttgatatgctgcctgctgacgctcttgacgattttgaccttgacatgctccccgggtaactaagtaaggatcaacatcgaattcgatttctgttcctgttaatcaacctctggattacaaaatttgtgaaagattgactggtattcttaactatgttgctccttttacgctatgtggatacgctgctttaatgcctttgtatcatgctattgcttcccgtatggctttcattttctcctccttgtataaatcctggttgctgtctctttatgaggagttgtggcccgttgtcaggcaacgtggcgtggtgtgcactgtgtttgctgacgcaacccccactggttggggcattgccaccacctgtcagctcctttccgggactttcgctttccccctccctattgccacggcggaactcatcgccgcctgccttgcccgctgctggacaggggctcggctgttgggcactgacaattccgtggtgttgtcggggaagctgacgtcctttccatggctgctcgcctgtgttgccacctggattctgcgcgggacgtccttctgctacgtcccttcggccctcaatccagcggaccttccttcccgcggcctgctgccggctctgcggcctcttccgcgtcttcgccttcgccctcagacgagtcggatctccctttgggccgcctccccgcctgtttcgcctcgggctcaatcactagtgaattcgataaaataaaagattttatttagtctccagaaaaaggggggaatgaaagaccccacctgtaggtttggcaagctagcttaagtaacgccattttgcaaggcatggaaaaatacataactgagaatagagaagttcagatcaaggtcaggaacagatggaacagctgaatatgggccaaacaggatatctgtggtaagcagttcctgccccggctcagggccaagaacagatggaacagctgaatatgggccaaacaggatatctgtggtaagcagttcctgccccggctcagggccaagaacagatggtccccagatgcggtccagccctcagcagtttctagagaaccatcagatgtttccagggtgccccaaggacctgaaatgaccctgtgccttatttgaactaaccaatcagttcgcttctcgcttctgttcgcgcgcttctgctccccgagctcaataaaagagcccacaacccctcactcggggcgccagtcctccgattgactgagtcgcccgggtacccgtgtatccaataaaccctcttgcagttgcatccgacttgtggtctcgctgttccttgggagggtctcctctgagtgattgactacccgtcagcgggggtctttcatttgggggctcgtccgggatcgggagacccctgcccagggaccaccgacccaccaccgggaggtaagctggctgcctcgcgcgtttcggtgatgacggtgaaaacctctgacacatgcagctcccggagacggtcacagcttgtctgtaagcggatgccgggagcagacaagcccgtcagggcgcgtcagcgggtgttggcgggtgtcggggcgcagccatgacccagtcacgtagcgatagcggagtgtatactggcttaactatgcggcatcagagcagattgtactgagagtgcaccatatgcggtgtgaaataccgcacagatgcgtaaggagaaaataccgcatcaggcgctcttccgcttcctcgctcactgactcgctgcgctcggtcgttcggctgcggcgagcggtatcagctcactcaaaggcggtaatacggttatccacagaatcaggggataacgcaggaaagaacatgtgagcaaaaggccagcaaaaggccaggaaccgtaaaaaggccgcgttgctggcgtttttccataggctccgcccccctgacgagcatcacaaaaatcgacgctcaagtcagaggtggcgaaacccgacaggactataaagataccaggcgtttccccctggaagctccctcgtgcgctctcctgttccgaccctgccgcttaccggatacctgtccgcctttctcccttcgggaagcgtggcgctttctcatagctcacgctgtaggtatctcagttcggtgtaggtcgttcgctccaagctgggctgtgtgcacgaaccccccgttcagcccgaccgctgcgccttatccggtaactatcgtcttgagtccaacccggtaagacacgacttatcgccactggcagcagccactggtaacaggattagcagagcgaggtatgtaggcggtgctacagagttcttgaagtggtggcctaactacggctacactagaaggacagtatttggtatctgcgctctgctgaagccagttaccttcggaaaaagagttggtagctcttgatccggcaaacaaaccaccgctggtagcggtggtttttttgtttgcaagcagcagattacgcgcagaaaaaaaggatctcaagaagatcctttgatcttttctacggggtctgacgctcagtggaacgaaaactcacgttaagggattttggtcatgagattatcaaaaaggatcttcacctagatccttttaaattaaaaatgaagttttaaatcaatctaaagtatatatgagtaaacttggtctgacagttaccaatgcttaatcagtgaggcacctatctcagcgatctgtctatttcgttcatccatagttgcctgactccccgtcgtgtagataactacgatacgggagggcttaccatctggccccagtgctgcaatgataccgcgagacccacgctcaccggctccagatttatcagcaataaaccagccagccggaagggccgagcgcagaagtggtcctgcaactttatccgcctccatccagtctattaattgttgccgggaagctagagtaagtagttcgccagttaatagtttgcgcaacgttgttgccattgctgcaggcatcgtggtgtcacgctcgtcgtttggtatggcttcattcagctccggttcccaacgatcaaggcgagttacatgatcccccatgttgtgcaaaaaagcggttagctccttcggtcctccgatcgttgtcagaagtaagttggccgcagtgttatcactcatggttatggcagcactgcataattctcttactgtcatgccatccgtaagatgcttttctgtgactggtgagtactcaaccaagtcattctgagaatagtgtatgcggcgaccgagttgctcttgcccggcgtcaacacgggataataccgcgccacatagcagaactttaaaagtgctcatcattggaaaacgttcttcggggcgaaaactctcaaggatcttaccgctgttgagatccagttcgatgtaacccactcgtgcacccaactgatcttcagcatcttttactttcaccagcgtttctgggtgagcaaaaacaggaaggcaaaatgccgcaaaaaagggaataagggcgacacggaaatgttgaatactcatactcttcctttttcaatattattgaagcatttatcagggttattgtctcatgagcggatacatatttgaatgtatttagaaaaataaacaaataggggttccgcgcacatttccccgaaaagtgccacctgacgtctaagaaaccattattatcatgacattaacctataaaaataggcgtatcacgaggccctttcgtcttcaagaattcataccagatcaccgaaaactgtcctccaaatgtgtccccctcacactcccaaattcgcgggcttctgcctcttagaccactctaccctattccccacactcaccggagccaaagccgcggcccttccgtttctttgct
